# Phylogenomics of two ST1 antibiotic-susceptible non-clinical *Acinetobacter baumannii* strains reveals multiple lineages and complex evolutionary history in global clone 1

**DOI:** 10.1099/mgen.0.000705

**Published:** 2021-12-07

**Authors:** Jonathan Koong, Claire Johnson, Rayane Rafei, Monzer Hamze, Garry S. A. Myers, Johanna J. Kenyon, Allison J. Lopatkin, Mohammad Hamidian

**Affiliations:** ^1^​ The iThree Institute, University of Technology Sydney, Ultimo, NSW, Australia; ^2^​ Department of Biology, Barnard College Affiliated Faculty Data Science Institute, Columbia University Affiliated Faculty, Columbia University, Columbia, USA; ^3^​ Laboratoire Microbiologie Santé et Environnement (LMSE), Doctoral School of Science & Technology, Faculty of Public Health, Lebanese University, Tripoli, Lebanon; ^4^​ Centre for Immunology and Infection Control, School of Biomedical Sciences. Faculty of Health, Queensland University of Technology, Brisbane, Australia

**Keywords:** *Acinetobacter baumannii*, global clone, GC1, ST1, antibiotic resistance, whole genome sequence

## Abstract

*
Acinetobacter baumannii* is an opportunistic pathogen that is difficult to treat due to its resistance to extreme conditions, including desiccation and antibiotics. Most strains causing outbreaks around the world belong to two main global lineages, namely global clones 1 and 2 (GC1 and GC2). Here, we used a combination of Illumina short read and MinION (Oxford Nanopore) long-read sequence data with a hybrid assembly approach to complete the genome sequence of two antibiotic-sensitive GC1 strains, Ex003 and Ax270, recovered in Lebanon from water and a rectal swab of a cat, respectively. Phylogenetic analysis of Ax270 and Ex003 with 186 publicly available GC1 genomes revealed two major clades, including five main lineages (L1–L5), and four single-isolate lineages outside of the two clades. Ax270 and Ex003, along with AB307-0294 and MRSN7213 (both predicted antibiotic-susceptible isolates) represent these individual lineages. Antibiotic resistance islands and transposons interrupting the *comM* gene remain important features in L1–L5, with L1 associated with the AbaR-type resistance islands, L2 with AbaR4, L3 strains containing either AbaR4 or its variants as well as Tn*6022*::ISAba42, and L4 and L5 associated with Tn*6022* or its variants. Analysis of the capsule (KL) and outer core (OCL) polysaccharide loci further revealed a complex evolutionary history probably involving many recombination events. As more genomes become available, more GC1 lineages continue to emerge. However, genome sequence data from more diverse geographical regions are needed to draw a more accurate population structure of this globally distributed clone.

## Data Summary

Whole genome sequences for a total of 186 non-redundant genome sequence data of *Acinetobacter baumannii* isolates predicted to represent strains that belong to global clone 1 were identified amongst 4797 *A *. *baumannii* genome assemblies publicly available in the GenBank non-redundant and Whole Genome Shotgun databases as of mid-November 2020. All genomes were retrieved and analysed with the genome sequences of two new GC1 strains that were completed in this study (188 genomes in total). The full strain list and the ftp addresses used to retrieve the genomes are publicly available at https://www.ncbi.nlm.nih.gov/genome/?term=Acinetobacter+baumannii.

Genomes completed as part of this study are available in GenBank under accession numbers CP049314–CP049315 (Ex003 chromosome and pEx003) and CP049240 (Ax270). The authors confirm all supporting data, code and protocols have been provided within the article or through supplementary data files

**Impact Statement**

*
Acinetobacter baumannii* continues to be a problematic human pathogen due to high levels of antibiotic resistance leading to treatment failures. Despite the significance of studying antibiotic-susceptible strains in determining the population structure, developing antibiotic resistance, and to predict future emerging clones, there have only been limited opportunities to analyse such strains as most genome sequence data represent multiply, extensively and pan-drug-resistant isolates. Furthermore, most publicly available genomes are predominantly from a subset of countries, leaving a substantial knowledge gap. Here, we completed the genome sequence of two antibiotic-sensitive *A. baumannii* strains belonging to global clone 1 (GC1) that were recovered from the environment, and we placed them in a global context, providing an overall snapshot of the evolution of GC1. Here, we show that GC1 isolates belong to multiple lineages, including five major antibiotic-resistant lineages that contained the largest number of strains carrying several antibiotic resistance genes, and additional lineages represented by antibiotic-susceptible or predicted to be susceptible strains. Combined evolutionary forces have highly diversified GC1 isolates and created multiple globally distributed lineages, subclones as well as locally evolved small groups limited to certain areas. This diversity reflects a complex picture that has arisen from a high degree of horizontal gene transfer impacting many genomic features such as antibiotic resistance gene repertoire (including lineage-specific resistance islands), sequence types and surface polysaccharides. These events also provide an opportunity to differentiate closely related strains and to dissect microevolutionary events, as well as predict paths that might lead to the emergence of future multi-resistant clones.

## Introduction

*Acinetobacter baumannii* is a Gram-negative opportunistic pathogen that causes a wide range of infections including pneumonia, and blood, wound and urinary tract infections [1, 2]. *A. baumannii* is one of the main causes of antibiotic-resistant infections in intensive care units (ICUs) with immunocompromised patients globally. It has emerged as an important superbug because of its high levels of resistance to many antibiotics including carbapenems [3–5]. Strains causing infections often belong to a limited number of clones with most multiply, extensively and pan-drug-resistant strains belonging to two globally distributed clones, namely global clones 1 and 2 (GC1 and GC2, respectively) [3–7].

The study of bacterial pathogens has been revolutionized in recent decades because of advances in whole-genome sequencing (WGS) technologies and bioinformatic tools, enabling gene screening and phylogenomic analysis of outbreak strains with unprecedented resolution [8, 9]. To date, over 4700 (as of early 2021) *A. baumannii* genomes have been sequenced and deposited in GenBank public databases. However, we recently showed that despite the global distribution of *A. baumannii* and the need to have data from all countries, there is a substantial gap in genome sequence data from most geographical regions across the globe [4], particularly isolates in GC1, and more specifically lineage 2 within GC1, which probably originated from the Middle East region [10, 11]. This region is of importance as it has been suggested that the return of soldiers from combat zones in the Middle East region is an important factor contributing to the rise and epidemiology of *A. baumannii* infections in the USA since 2000 [12, 13].

Another factor limiting the ability to study the genomic evolution and population structure of successful multi-drug-resistant clones at the global level, and to further understand how antibiotic resistance develops and spreads, is the study of both antibiotic-susceptible strains and non-clinical strains that belong to the major global clones. However, to date, antibiotic-sensitive strains have received little attention; to the best of our knowledge, amongst >4700 publicly available *A. baumannii* genomes, there is only a single genome from an antibiotic-sensitive GC1 strain, AB307-094 (GenBank accession number CP001172.2), that has been studied in detail.

Here, we completed and analysed the genome sequences of two further antibiotic-susceptible GC1 isolates that were recovered from non-clinical sources in Lebanon and show that they are closely related to the antibiotic-resistant members of this globally distributed clone. To place these two genomes in the greater GC1 context, we re-visit the phylogenetic relationship of all GC1 genomes using the two Lebanese isolates and those publicly available in GenBank non-redundant and Whole Genome Shotgun databases (as of early 2021).

## Methods

### Bacterial isolates sequenced in this study

Ex003 was isolated in 2012 from a water sample of an artesian well in Koura, North Lebanon, in a previous study of non-clinical *A. baumannii* isolates in Lebanon where it was identified at the species level [14].

Ax270 was isolated from a rectal swab of a cat in 2015 in Beirut, Lebanon, and confirmed to be *A. baumannii* as part of a nationwide study to isolate *Acinetobacter* species [15].

### Antibiotic susceptibility testing

For Ex003 and Ax270, the antibiotic resistance profile against 22 antibiotics was determined by disc diffusion using the calibrated dichotomous sensitivity disc diffusion method (CDS) (http://cdstest.net), as previously described [16]. Antibiotics tested were: streptomycin (25 µg), spectinomycin (25 µg), sulfamethoxazole (100 µg), trimethoprim (5 µg), kanamycin (30 µg), neomycin (30 µg), gentamicin (10 µg), amikacin (30 µg), tobramycin (10 µg), netilmicin (30 µg), rifampicin (30 µg), tetracycline (30 µg), ampicillin (25 µg), ampicillin/sulbactam (10/10 µg), cefotaxime (30 µg), ceftazidime (30 µg), imipenem (10 µg), meropenem (10 µg), piperacillin/tazobactam (100–10 µg), timentin (ticarcillin/clavulanic acid) (75/10 µg), ciprofloxacin (5 µg) and nalidixic acid (30 µg). Resistance and susceptibility were interpreted according to the Clinical and Laboratory Standards Institute (CLSI) guidelines for *Acinetobacter* species [17] and CDS method when a CLSI breakpoint for *Acinetobacter* species was not available (e.g. for netilmicin, streptomycin, spectinomycin, sulfamethoxazole, nalidixic acid, and rifampicin).

### Whole genome sequencing and sequence analysis

Whole-cell genomic DNA was isolated, using the DNeasy Microbial Kit (Qiagen), from cells grown overnight at 37 °C in Luria Broth inoculated from a frozen stock derived from a single colony. The DNA was subjected to library preparation and barcoding for Illumina MiSeq and MinION (Oxford Nanopore Technologies) sequencing as described in detail previously [18]. Both Illumina MiSeq and MinION sequencing were done in-house at the University of Technology, Sydney, Australia, generating 41 737 and 34 255 paired-end short reads with an average length of 250 bp and a total of 3104 and 3141 MinION reads for both Ex003 and Ax270, respectively. The FastQC (v.0.11.9) program (https://bioinformatics.babraham.ac.uk/projects/fastqc/) was used (default settings) to check the quality of Illumina reads (e.g. per-base sequence quality of >38, per-base sequence content of <10 %, etc.). Filtlong (v.0.2.0) (https://github.com/rrwick/Filtlong) was used to filter the MinION reads with low quality by discarding any read shorter than 1 kbp (--min_length 1000) and the worst 10 % of reads is measured by base pairs (--keep_percent 90). The Illumina and MinION reads were assembled *de novo* using a hybrid assembly approach using the Unicycler (v0.4.8) program publicly available at https://github.com/rrwick/Unicycler [19], generating an average 100-fold final genome coverage for both genomes. The final assembly was annotated automatically using Prokka (v.1.13) [20] followed by manual annotation of all regions of interest including using tools as detailed below.

To examine the phylogenomic relationship of Ex003 and Ax270 to other members of GC1, all 4797 *A *. *baumannii* genomes available in the GenBank non-redundant and Whole Genome Shotgun databases (as of mid-November 2020) were downloaded. Multi-sequence sequence types (MLSTs) were determined using the mlst software (T. Seemann, mlst Github https://github.com/tseemann/mlst), which uses the *A. baumannii* PubMLST database (available at https://pubmlst.org/organisms/acinetobacter-baumannii). Both the Institut Pasteur [21] and Oxford types [22] were determined. GC1s were defined as *A. baumannii* genomes that belong to sequence type ST1 in the Pasteur scheme, as well as its single and double locus variants. Briefly, previously sequenced genomes, and their associated metadata, were downloaded from the NCBI FTP server in November 2020. Genomes were first filtered for quality (number of contigs <250 and N50 >25 000). The remaining high-quality genomes that did not belong to clonal group 1 (Table S2), as determined by MLST, were further discarded. Finally, any duplicate (found by matching strain names and country and date of isolation) genomes were filtered for the one with the greatest level of completion, resulting in a set of 188 final clonal group 1 genomes (including Ax270 and Ex003) for comparison (Table S2).

The presence of antibiotic resistance genes and insertion sequences were examined in all GC1 genomes using the Abricate software v.0.8.10 (T. Seemann, Abricate, Github https://github.com/tseemann/abricate), which uses the ResFinder (https://cge.cbs.dtu.dk/services/ResFinder/) database. Insertion sequences in regions of interest were found using the ISFinder (https://www-is.biotoul.fr/) program search tools. Genomic loci for variable surface polysaccharides were also typed by identifying genes at the K locus (KL) for capsular polysaccharide biosynthesis and the OC locus (OCL) for synthesis of the outer core (OC) of the lipooligosaccharide using Kaptive v 0.7 with *A. baumannii* KL and OCL reference databases [23], respectively. Novel KL and OCL types were manually inspected and annotated using the established nomenclature system as described previously [23, 24].

### Phylogenetic analysis

A maximum-likelihood phylogenetic tree was reconstructed from a core genome alignment using short read data. The *readSimulator* program (https://github.com/wanyuac/readSimulator) was used to generate artificial short read data of genomes for which read data were not publicly available in GenBank. Briefly the sequence reads for all isolates were mapped to the *A. baumannii* A1 strain, which was used as a reference (GenBank accession no. CP010781) using the Snippy software (v.2.0) (available at https://github.com/tseemann/snippy) to generate a whole-genome alignment. High-quality variant sites were called using SAMtools (v1.3.1.24) as described previously [11]. Single nucleotide differences (SNDs) located in recombinant regions were identified and removed using the Gubbins (v2.1.025) program [25] with default parameters, including a default taxon filtering percentage of 25 %. A maximum-likelihood phylogenetic tree was inferred from the resulting alignment (a total of 55 051 non-recombinant SNPs) using RAxML (v.8) with the generalized time reversible (GTR) Gamma model of nucleotide substitution [26]. Ten independent runs of RAxML with 100 bootstraps each gave near identical results. The GC2 strain A320 (RUH134), recovered in 1982 in the Netherlands (GenBank accession number CP032055), was used as the outgroup for phylogenetic analysis, and subsequently removed from the tree to facilitate visualization. Lineage and sub-lineage assignments found by phylogenetic analysis were further confirmed using the fast BAPS algorithm [Fast hierarchical Bayesian analysis of population structure (fastbaps); https://github.com/gtonkinhill/fastbaps], which applies the hierarchical Bayesian clustering (BHC) algorithm to determine clusters of multi-sequence genotypes. Fastbaps clusters were determined using the optimised.symmetric prior setting as previously described [27]. The phylogenetic tree along with additional features were visualized and plotted using the ggplot2 (v.3.3.5) [28] and ggtree [29] packages in R (v.4.0.5).

## Results

### Identification of two non-clinical GC1 isolates susceptible to most clinically used antibiotics

Antibiotic susceptibility profiles of two non-clinical *A. baumannii* isolates, Ax270 and Ex003, described in a previous study [15] and found to belong to GC1 [30], were determined here. Amongst the 22 antibiotics tested using the CDS method, Ax270 only showed resistance to nalidixic acid and reduced susceptibility to trimethoprim (Table S1). Ex003 exhibited reduced susceptibility only to trimethoprim. All *A. baumannii* strains are known to be intrinsically resistant to chloramphenicol and, consistently, Ax270 and Ex003 were no exception (Table S1). Thus, Ax270 and Ex003 were classified as antibiotic-susceptible isolates.

### Complete genome sequences of Ax270 and Ex003

To assess the genetic relationship of both Ax270 and Ex003 to other GC1 isolates within the global context, the whole genome sequences for Ax270 and Ex003 were obtained by a combination of Illumina and MinION sequencing and assessed using automated (Prokka) and manual (using Pfam and ISFinder search tools) methods. The complete genome of Ax270 includes a 3 764 882 bp chromosome but contains no plasmids, whereas the complete genome of Ex003 contains a 3 889 729 bp chromosome and an ~11 kbp cryptic plasmid (see section below). Examination of the WGS data revealed that Ex003 and Ax270 belong to ST1_IP_ (Institut Pasteur scheme) and ST231_OX_ (Oxford scheme), and both encode the OXA-69 variant of the intrinsic *oxaAb* gene, consistent with their assignment [6, 7] to GC1. Both genomes also encode a KL1 capsular polysaccharide biosynthesis locus and an OCL1 outer core biosynthesis locus, allowing the strain designation of ST1:ST231:KL1:OCL1, which is believed to be representative of the ancestral GC1 type [11].

Ex003 and Ax270 did not contain any antibiotic resistance genes. However, in Ax270, resistance to fluoroquinolones was found due to the mutations found in the *gyrA* DNA gyrase and *parC* topoisomerase IV genes, leading to GyrA S81→L81 and ParC S84→L84 substitutions. These specific mutations are well known to cause resistance to fluoroquinolones in Gram-negative bacteria including *A. baumannii* (e.g. nalidixic acid) [31, 32].

In many *A. baumannii* GC1 genomes, the chromosomal *comM* gene is often interrupted by variants of AbaR-type resistance islands [6]. Interestingly, the *comM* gene in the Ax270 genome is intact. For Ex003, *comM* was found to be interrupted by a 14 032 bp insertion (bases 3 641 288–3 655 319 of GenBank accession number CP049314) that encodes 10 ORFs including XerC/E recombination proteins (locus_id G5571_03465–66), a RelA guanosine pentaphosphate (locus_id G5571_03470) and helicase (locus_id G5571_03471). Notably, this 14 kbp insertion was found between bases 901 and 902 of *comM* while AbaRs are often present between bases 835 and 836 indicating a different insertion point compared to AbaRs. It was also found to be a novel sequence segment by searching the GenBank non-redundant database, which only retrieved a single blast hit of only 2166 bp with 75 % DNA identity in *Acinetobacter bereziniae* strain GD03185 (GenBank accession number CP066119) spanning an ORF (locus_id G5571_03469) and the *relA* gene (locus_id G5571_03470). Careful examination of the *comM* gene and its boundaries with the inserted segment to find properties of transposons or genomic islands, including target site duplication and/or any inverted repeats flanking the insertion, that could explain how insertion occurred was inconclusive (Fig. S1, available in the online version of this article). Therefore, we speculate that this segment is likely to be a remnant of an ancient insertion where its ends have been deleted due to unclear events. This could not be pursued further as sufficient genome sequence data are currently unavailable.

### Ex003 carries a novel cryptic plasmid

**Fig. 1.**
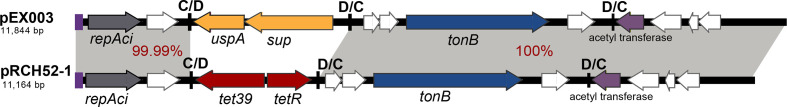
Linearized map of pEx003 and its comparison to pRCH52-1. Central thick grey arrows show the plasmid backbones with horizontal arrows indicating the extent and orientation of genes/ORFs. Vertical black bars show p*dif* sites and vertical purple bars at the beginning of each plasmid indicate the location of iterons. This figure was drawn to scale in Inkscape v.0.92 using sequences in GenBank accession numbers CP049315 (pEx003) and KT346360.1 (pRCH52-1).

Ex003 contains an 11 844 bp cryptic plasmid called pEx003 (Fig. 1). pEx003 encodes a putative replication initiation protein (Rep) identical to that encoded by pRCH52-1 (GenBank accession number KT346360) found in *A. baumannii* RCH52: an ST729_IP_ strain that belongs to the European clone III [33]. The closest known Rep to those encoded by pEX003 and pRCH52-1 is RepAci7 (GenBank accession number GU978996), with 96 % amino acid identity.

Recently, several *A. baumannii* plasmids, including pRCH52-1, have been shown to include specific modules, called p*dif* modules, flanked by p*dif* sites, consisting of inversely orientated binding sites for the XerC and XerD recombinases separated by 6 bp [34–37]. Analysis of pEx003 showed that it is almost identical to pRCH52-1 except that the *tet39* module (found in pRCH52-1) is replaced with another p*dif* module encoding a universal stress protein (*uspA*) and a sulphate permease (*sup*) (Fig. 1). Searching the GenBank non-redundant database, to find the *uspA-sup* module, revealed several homologue modules with DNA identities ranging from 75 to 98 %. These modules are widely spread in many *Acinetobacter* plasmids, indicating that it belongs to a diverse family. This was not pursued further.

### GC1 includes multiple lineages: insights from phylogenomics of Ax270 and Ex003

**Fig. 2.**
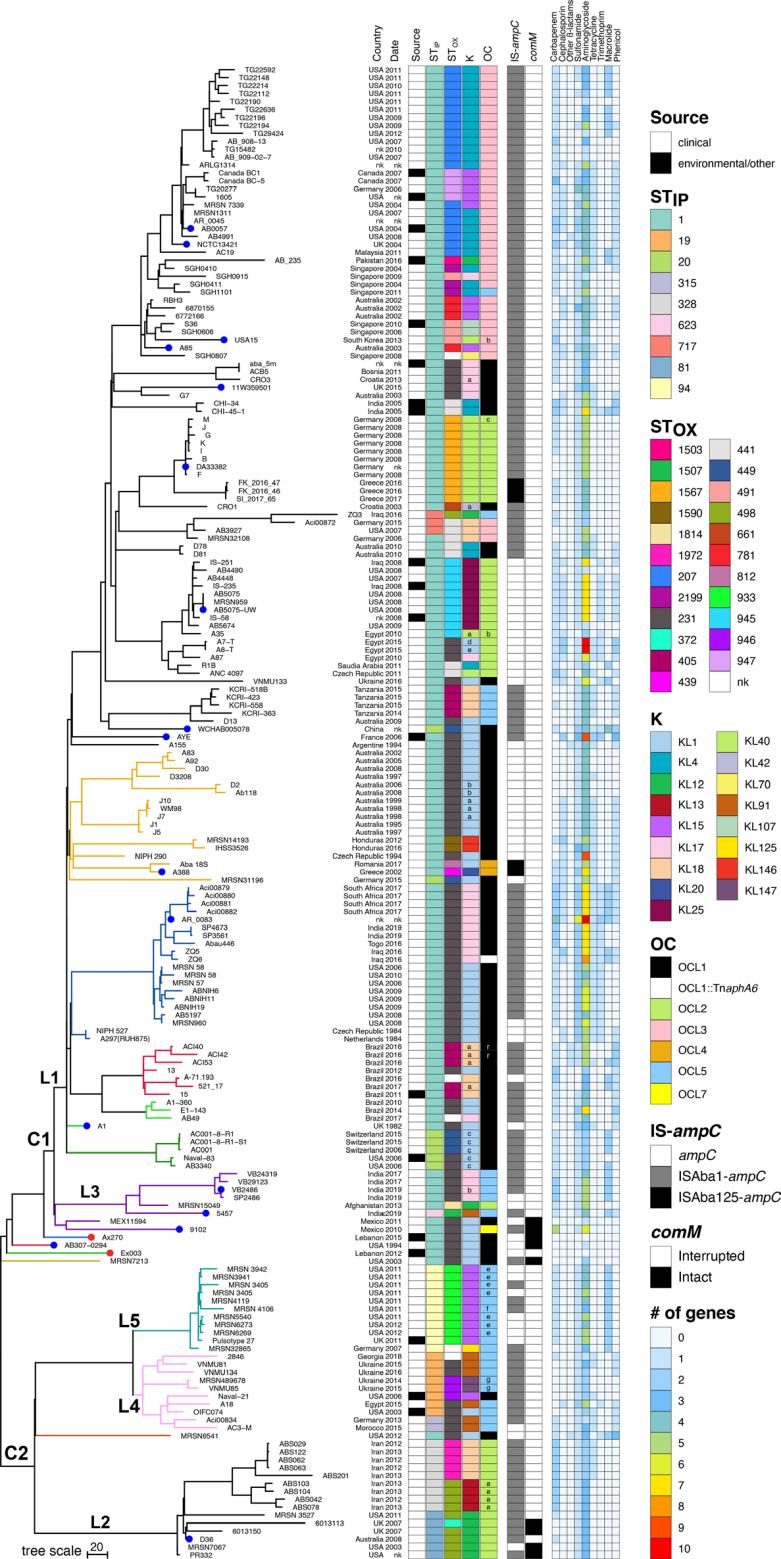
Phylogenetic tree of all publicly available GC1 strains. Filled nodes indicate complete genomes with Ax270 and Ex003 coloured red. The right-hand panel (from left to right) contains metadata, isolation date, isolation source, ST_IP_, ST_OX_, K, OC, the presence/absence of IS (insertion sequence) upstream of the chromosomal *ampC* gene, whether the *comM* gene is interrupted and the number of antibiotic resistance genes present with antibiotic families indicated above. Main clades, C1 and C2, and lineages L1–5 are shown using uppercase letters and numbers. All BAPS groups (lineages and sublineages) are shown with their branches coloured. Minor variants of each K/OC type are indicated using a small letter on the box.

To place Ax270 and Ex003 in the broader GC1 phylogeny, all publicly available *A. baumannii* genomes (>4700 genomes as of mid-November 2020) were downloaded and screened for GC1s using the criteria specified previously (known STs such as ST1_IP_ and its single- and double-locus variants) [11]. In total, 186 non-redundant GC1 genomes were found (including 19 complete genomes), all encoding OXA-69 variants of the *oxaAb* gene, which is consistent with their assignment to GC1 [11]. With these genomes together with Ax270 and Ex003, 188 assemblies were used to construct a core genome phylogeny of all available GC1 genomes (Fig. 2).

In previous studies using a smaller collection of GC1 genomes [10, 11], the GC1 population structure was shown to consist of two main lineages, lineage 1 (L1) and lineage 2 (L2). L1 included a geographically and genetically diverse set of strains, whereas L2 was more homogeneous and associated with strains isolated in the Middle East or linked to this region via isolates recovered from soldiers or military personnel returning from the Middle East region. Recently, two studies proposed that four Indian isolates (VB24319, VB29123, VB2486 and 5457) might belong to a third lineage, designated L3 [38, 39].

The core genome phylogeny constructed here using a total of 188 GC1 genome assemblies again reveals two major clades (designated here as C1 and C2), and additional lineages with only one member (Fig. 2). Both C1 and C2 consist of several main lineages, some with additional sub-lineages, which were further confirmed using the fastbaps approach (Fig. S2, and coloured branches in Fig. 2). Clade 1 includes the largest number of isolates (*n*=147) and consists of the originally identified lineage 1 (L1) and lineage 3 (L3).

L1 contains seven distinct sub-lineages (BAPS groups). In L1, a large variety of geographical regions are represented, though 14 are from non-clinical, environmental or other sources. Isolates that fall into this lineage predominantly belong to ST1_IP_ but exhibit more variety in the ST_OX_ types, probably reflecting a difference in the sequence for the *gpi* allele that sits within the K locus for capsular polysaccharide synthesis (Fig. 2). Interestingly, A1, which is the earliest (recovered in 1982 in the UK) GC1 available [11, 40], and used as the reference strain, appears to form its own group (in both the core genome phylogeny and fastbaps tree).

The four Indian isolates forming the previously proposed L3 lineage [38] cluster with two additional isolates not previously examined: SP2486 (recovered in India in 2019) and MRSN15049 (recovered in Afghanistan in 2013). The four Indian isolates have the same ST_IP_:ST_OX_:KL:OCL profiles, and establish the designation of L3 as the third major lineage of GC1 (Fig. 2), although MRSN15049 and 5457 appear to have further diverged as they include differences in their ST_IP_:ST_OX_:KL:OCL profile (Fig. 2). In addition, we previously showed that 5457 carries Tn*6171*-v1, which is a variant of a Tn*7*-family transposon that carries genes for synthesis and uptake of the fimsbactin siderophore system, with other L3 members (VB24319, VB29123 and VB2486) carry another variant called Tn*6171*-v2 [38]. Here, we show that SP2486, which clusters with VB24319, VB29123 and VB2486, also carries the same variant, Tn*6171*-v2. However, MRSN15049 (recovered in Afghanistan) does not carry Tn*6171* or a variant of Tn*6171*, indicating that it has evolved differently and that the acquisition of fimsbactin encoding transposons is associated with L3 strains recovered in India. Two additional strains, 9102 and MEX11594 (both recovered in Mexico in 2010 and 2011, respectively), are further divergent (Fig. 2) but also belong to L3 (confirmed by fastbaps analysis).

Compared to clade 1, clade 2 consists of a smaller number of isolates but similarly represents a diverse set (Fig. 2). This clade has previously been referred to as lineage 2, which was described as including an ST81_IP_/ST328_IP_ sublineage associated with military sources or the Middle East region and a second sublineage with ST19_IP_ and ST94_IP_ isolates [38]. Here, our phylogenetic and fastbaps analysis identified a total of three major lineages (here referred to as L2, L4 and L5) in C2. The name L2 is retained for the ST81_IP_/ST328_IP_ lineage, which is differentiated from L4 and L5 by the acquisition of AbaR4 in the *comM* gene. L4 and L5 represent ST19_IP_ and ST94_IP_ isolates, respectively. However, here we also identify two isolates, Aci00834 and AC3-M, of an additional ST_IP_, ST315_IP_, in L4. This might represent a recent change and that these two strains are likely to form a new lineage in future. A single ST1_IP_ isolate, TG19582, was also previously shown to belong to the original L2 lineage. However, on re-examination, TG19582 was not included in this phylogenomic analysis due to sequence quality cut-offs. A new ST1_IP_ isolate, MRSN6541, forms a separate lineage within C2 (Fig. 2), probably representing an emerging, under sampled lineage.

Four GC1 genomes, AB307-0294, MRSN7213, Ax270 and Ex003, do not cluster with the five main lineages. Previous studies have noted that one of these isolates, the antibiotic-susceptible strain AB307-0294 (from a patient hospitalized at Erie County Medical Centre, Buffalo, NY, in 1994 [41]; GenBank accession no. CP001172.1) could not be accurately assigned to a lineage because of uncertainty with the genome quality as the original genome was sequenced using pyrosequencing technology [11]. The genome sequence of AB307-0294 has since been updated (GenBank accession no. CP001172.2) and was included here in the reconstructed GC1 phylogeny. Again, our analysis shows that AB307-0294 does not belong to either L1, L2, L3, L4 or L5. The three other isolates, Ax270, Ex003 and MRSN7213 (recovered in the USA in 2003), are also antibiotic-susceptible and each also represents a separate lineage with currently only one member available.

### Complex antibiotic resistance evolutionary history

Most genomes included in the GC1 phylogeny are from isolates recovered from clinical samples. However, there are also isolates in L1, L4 and L5 that were recovered from non-clinical samples, confirming that GC1 isolates are also present outside hospital settings (Fig. 2). This enables an overall snapshot of the diversity in GC1 genomes to provide insights into the evolution of antibiotic resistance in this clone.

It is known that several mechanisms, including point mutations, mobile genetic elements (transposons, resistance islands, plasmids, etc.), IS-mediated activation of chromosomal genes and homologous recombination, have contributed to the evolution of antibiotic resistance in GC1 strains [3, 5–7, 11, 38, 42, 43]. Here, the analysis of acquired antibiotic resistance genes shows the accumulation of several genes in members of L1–L5, conferring resistance to the most clinically relevant antibiotic families, most importantly carbapenems, and aminoglycosides (Fig. 2, Table S2). Interestingly, the genomes of Ex003, Ax270, AB307-0294 and MRSN 7213, which do not belong to the five main lineages, contain no antibiotic resistance genes, except for MRSN 7213 which only contains a single gene for aminoglycoside resistance.

Previously it was shown that in L1 most acquired antibiotic resistance genes are located in variants of the AbaR resistance islands interrupting the chromosomal *comM* gene [6]. However, in L2 this gene is either intact or occupied by the AbaR4 carbapenem resistance island, which carries the *oxa23* carbapenem resistance island in an ISAba1-bounded transposon called Tn*2006* [4, 10]. Regardless of source, only four L2 isolates, as well as Ax270, AB307-0294 and MRSN7213 strains representing antibiotic susceptible lineages, and 9102 and MEX11594 (both from Mexico in L3) carry an intact copy of the *comM* gene. In L4 and L5, *comM* is occupied with either Tn*6022*, which is the transposon backbone of AbaR4, or its deletion variants (Tn*6022*∆).

**Fig. 3.**
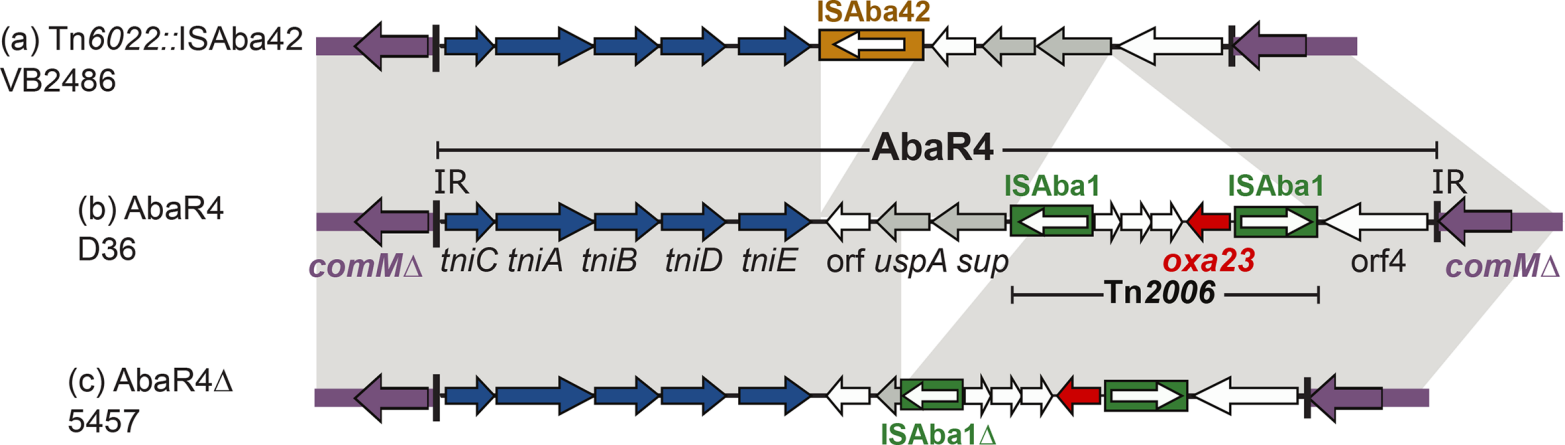
Genetic structure of transposons occupying the *comM* gene of complete L3 strains compared to D36. (**a**) The structure of Tn*6022*::ISAba42 found in *comM* in strain VB2486 (GenBank accession number CP050403.1); (**b**) AbaR4 found in *comM* of strain D36 (GenBank accession number CP012952.1); and (**c**) the structure of AbaR4∆ found in *comM* of strain 5457 (GenBank accession number CP045541.1). Horizontal arrows indicate the extent and direction of genes with transposition genes coloured dark blue, the *oxa23* carbapenem resistance gene red, and ORFs encoding unknown functions white. ISAba1 copies are shown using filled green boxes with white arrows (inside the boxes) indicating the orientation of their transposition genes . The extent of AbaR4 and Tn*2006* are also shown using thin horizontal boxes.

As L3 is a recently described lineage, insertions in the *comM* gene from the complete genomes of 5457 and VB2486 were closely examined. In 5457, the *comM* gene is occupied by a deletion variant of AbaR4 (AbaR4∆; Fig. 3). Compared to AbaR4, the deletion is 2506 bp removing the *sup* gene, a large fragment of *uspA* and 321 bp of ISAba1 (Fig. 3). It is not clear whether the deletion occurred before or upon insertion in *comM* as the transposition module is not impacted by this deletion. In VB2486, *comM* is interrupted with a Tn*6022* interrupted with an ISAba42 (Tn*6022*: ISAba42; Fig. 3). Analysis of the draft genomes of other Indian L3 strains predicted that SP2486 contains Tn*6022*::ISAba42 while others (VB24319, VB29123 and MRSN15049) might contain an AbaR4 in the *comM* gene as they contain all the AbaR4 segments and specific junctions. Furthermore, this indicates that AbaR4 and/or its variants have targeted the *comM* gene of GC1s on at least two occasions, interrupting the *comM* gene of (i) ST81/328 strains in L2 and (ii) Indian and Afghani strains in L3. Tn*6022*::ISAba42 also could have arisen either by direct insertion or acquisition of AbaR4 followed by ISAba42 interruption of the backbone and loss of Tn*2006*. Thus, this highlights that *comM* interruption is often an indication for the presence of an AbaR-type island in L1, AbaR4 in ST81/328 of L2, again AbaR4 or its variants in L3, and Tn*6022* (or its variants) in L4 and L5.

In *A. baumannii *, resistance to third-generation cephalosporins occurs via IS-mediated activation of the chromosomal *ampC* gene [43, 44]. Here, screening all GC1 genomes showed that ISAba1, or ISAba125 (in five strains: four from Greece and one Romanian strain), is present upstream of the *ampC* gene, presumably leading to third-generation cephalosporin resistance. However, it is notable that there are several sub-clades in all lineages with no IS preceding *ampC*, indicating that IS acquisition has occurred via multiple events and that the IS activation of *ampC* is not an early event in the history of GC1 (Fig. 2). It was previously established that in an ISAba1/ISAba125-activated *ampC* gene, along with its flanking chromosomal segments, can be acquired from an exogenous source via homologous recombination [45]. Here, examining the *ampC* sequences revealed several different sequences across the set, suggesting an important evolutionary role for homologous recombination in rendering GC1s to third-generation cephalosporin-resistant strains.

In general, carbapenem resistance is known to occur in *A. baumannii* predominantly via the oxacillin carbapenemases (OXA-23, OXA-58, OXA24, etc.) with metallo β-lactamases rarely seen [4]. Here, it was noted that amongst 188 genomes screened, 152 were predicted to contain an acquired oxacillin carbapenemase gene with *oxa23* (*n*=125) being by far the most widespread. As indicated, in L2, ST81/328 clade, *oxa23* is in Tn*2006*, in AbaR4, which occupies the *comM* gene. In other clades it is also often in either Tn*2006* or Tn*2006* in AbaR4, which could also be on RepAci6 plasmids [46]. However, 15 genomes, eight of which are from India, contained the *bla*_NDM_ metallo β-lactam carbapenem resistance genes, which is alarming as most were found to be on potentially conjugative plasmids.

### Variations at the KL for capsular polysaccharide synthesis

**Fig. 4.**
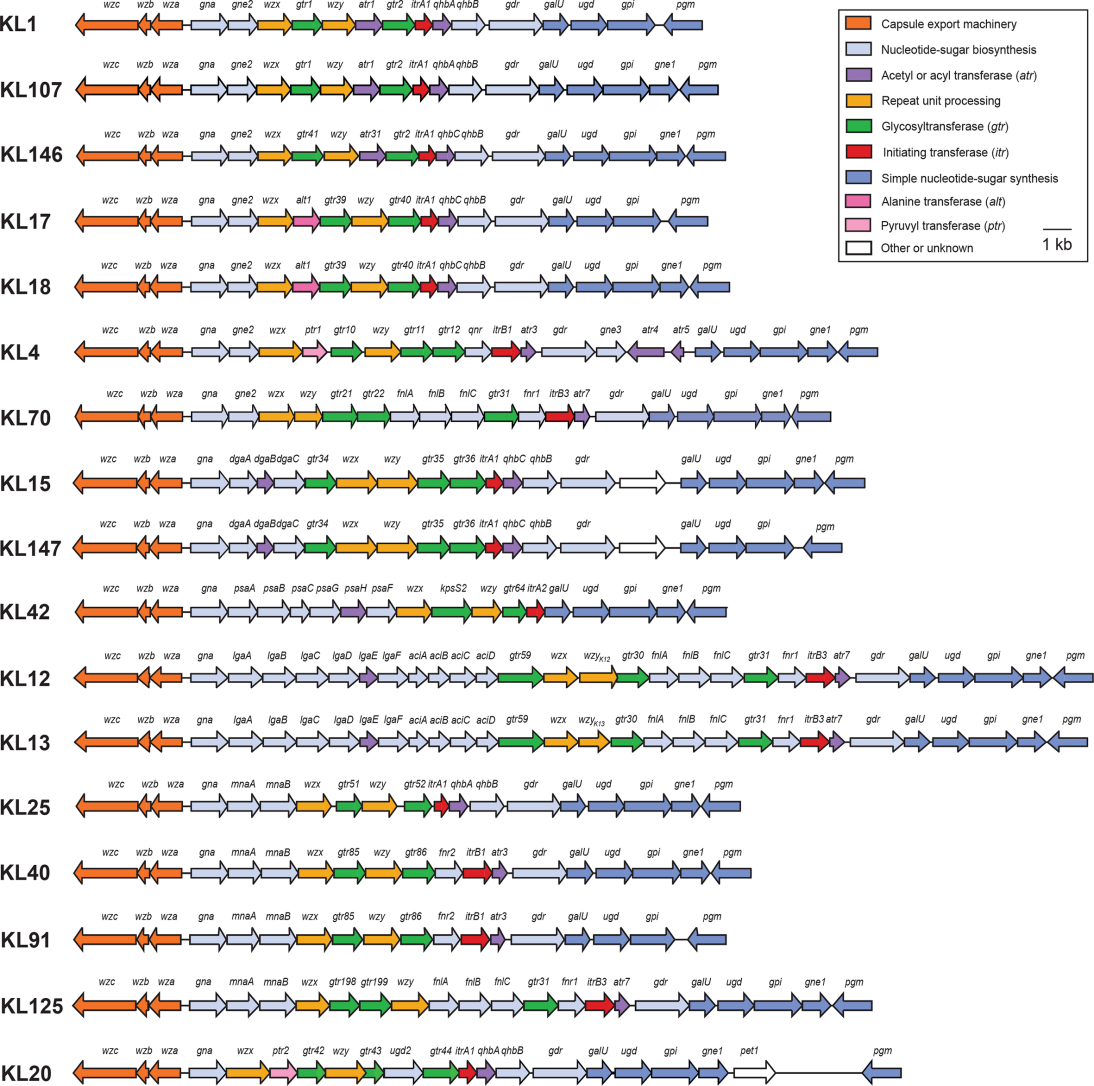
Gene clusters at the capsular polysaccharide K locus in GC1 genomes. Colours of genes represent categories of functions of the encoded products and the colour scheme is shown on the top right. Figure is drawn to scale.

One of the most variable regions in GC1 genomes, defined by SNP density and genetic content [11], is the KL responsible for synthesis of the capsular polysaccharide, a major virulence determinant. KL typing has proven useful for epidemiological studies that track the local and global dissemination of *A. baumannii* [10, 11, 47–49] and was similarly applied here. A total of 17 KL types (Fig. 4, Table S3) were identified amongst the 188 GC1 genomes examined, including five arrangements (KL70, KL91, KL125, and two novel KLs designated KL146 and KL147) not previously reported in GC1 isolates. Twenty genomes include variants of known types interrupted by an IS, which were designated using letters following the KL name to distinguish the variant type. Most KLs were found widely distributed across the GC1 phylogeny, though KL20, KL42, KL70 and KL125 were only in single isolates.

The KL1 gene cluster represents the most predominant KL type in the collection studied, found in 49 GC1 isolates recovered from different countries. KL1 was first reported in GC1 isolates, AYE and AB307-0294 [24], and is considered the likely ancestral KL type for GC1 [11]. It has also previously been shown to account for the largest proportion of ST1_IP_ genomes [23]. In the GC1 phylogeny, KL1 is distributed across the major L1, L3, L4 and L5 lineages and is also found in the genomes of all four antibiotic-susceptible strains (Fig. 2). Of the 49 genomes with KL1, 12 are variants named KL1a–KL1e that are found only in the L1 lineage.

### Variations at the OCL for synthesis of the lipooligosaccharide outer core

**Fig. 5.**
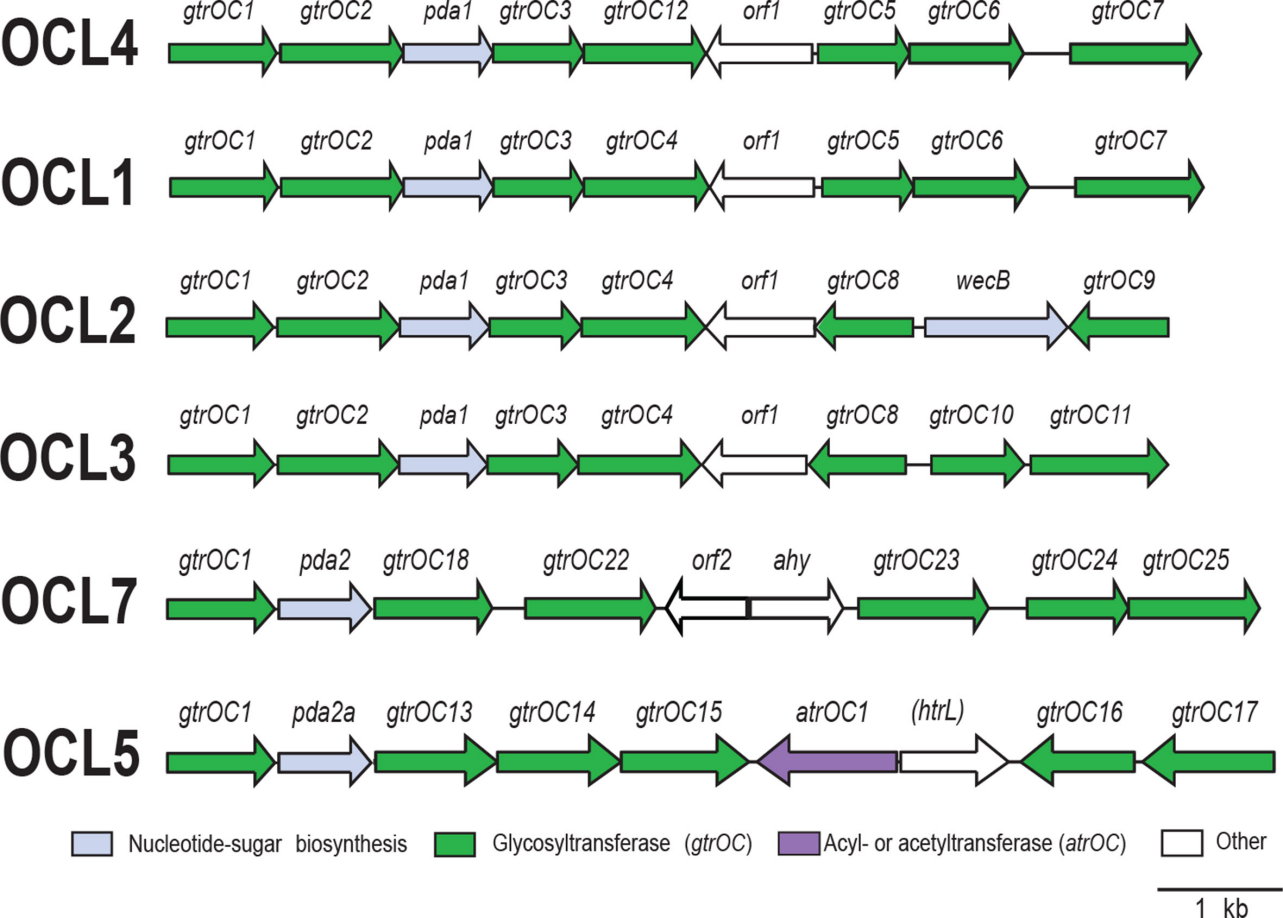
Gene clusters at the OCL for LOS (Lipooligosaccharide) outer core biosynthesis in GC1 genomes. Colours of genes represent categories of functions of the encoded products and the colour scheme is shown below. Figure is drawn to scale.

Another hypervariable region previously observed in GC1 genomes is the OCL directing synthesis of the outer core component of the lipooligosaccharide [11]. A total of six OCL types (Fig. 5, Table S3) were found in 188 GC1 genomes and all six types have previously been observed in GC1 [23]. OCL1 is the proposed ancestral type [11], and it was found here in 74 genomes, five of which carry one of three different variants of OCL1. One of these variants, OCL1b, has been previously described for the GC1 isolates, D78 and D81 [11]. The other two variants are novel. Two Brazilian isolates in the L1 lineage have OCL1r, which is interrupted by an IS inserted between *gtrOC5* and *gtrOC6*. The third variant is carried by the L1 isolate, ZQ6, recovered from Iraq. Interestingly, this variant includes a Tn*aphA6* transposon inserted between *gtrOC6* and *gtrOC7*. Tn*aphA6* is a composite ISAba125-bounded transposon that carries the *aphA6* amikacin, kanamycin and neomycin resistance gene and is widely present in *A. baumannii* strains [50, 51]. KL or OCL interruptions involving a larger transposon rather than an IS have not been reported for *A. baumannii *. Therefore, to distinguish this variant from others, the locus type was designated OCL1::Tn*aphA6*.

Of the remaining OCL types, OCL2 and OCL5 appear widespread in all three major lineages. OCL3 appears common within a sublineage of L1 ST1_IP_ genomes that also include KL4 or KL15. OCL4 is found in only two L1 isolates, A388 and Aba 18S, while OCL7 is in a single L3 isolate, 9102, from Mexico.

## Discussion

*A. baumannii* infections have high mortality rates of approximately 30–60 % mainly due to high levels of antibiotic resistance leading to treatment failure [52]. GC2 followed by GC1 contribute to the highest proportion of strains that cause multi-drug-resistant infections globally [4]. Over the last decade, studies have unravelled many complexities of: (i) how antibiotic resistance has developed in GC1 by providing the details of genetic mechanisms leading to antibiotic resistance [4, 6, 11, 53, 54], and (ii) characterizing variations in the K and OC surface polysaccharides [23, 24, 55]. Besides AB307-0294, all isolates studied previously are resistant to multiple antibiotics. AB307-0294 has been an important strain in helping better understand how resistance has developed in multi-resistant strains.

In this study, we have acquired two new antibiotic-susceptible GC1 isolates from Lebanon, Ax270 and Ex003, a region that has previously been underrepresented in epidemiological studies. Whole genome sequences for these isolates and those currently available in the public domain enabled an overall picture of the evolution of GC1 to be generated with a recombination-free phylogeny constructed with clinical and non-clinical isolates from different geographical regions and with varying antibiotic resistance profiles. A recent study examined diversity within the GC1 population, and proposed a new lineage, L3 [38, 39]. However, this phylogenetic analysis did not exclude nucleotide variants found in regions acquired by recombination and those in accessory genomes [38, 39] while inclusion of these variants often leads to a skewed tree topology given the high rate of horizontal gene transfer in GC1s. The addition of newly available GC1 genomes in our recombination-free phylogenetic tree revealed an expansion of established lineages (Fig. 2). This allowed the reassessment of isolates within each major clade, thus enabling the separation of three distinct major lineages within the original L2 lineage that we propose should be renamed as L2, L4 and L5, representing the additional major GC1 lineages. Our analysis also allowed us to define several distinct sublineages within L1. As more genome sequence data become available, it can therefore be envisaged that new lineages may begin to appear in the GC1 phylogenetic tree.

The GC1 phylogeny demonstrates that the differentiation of major lineages and sublineages within a clone cannot be guided by the ST_IP_ or ST_OX_ type alone. For example, each major lineage includes at least two ST_IP_ and several ST_OX_. However, inclusion of the KL and OCL type in combination with ST_IP_ and ST_OX_ can provide additional information on the relationship of an isolate to others within the global context. The combination of these four factors can be presented in a quick typing index as ST_IP_:ST_OX_:KL:OCL, as previously proposed [56]. For GC1, all susceptible isolates examined in this study, as well as many belonging to L1, have a quick typing index of ST1:ST231:KL1:OCL1. This index is also represented by two emerging strains involving MEX11594 and MRSN6541.

The complete phylogeny draws a complex picture of resistance to a range of antibiotic families (with many strains containing more than 10 antibiotic resistance genes) and surface polysaccharides. However, small branches tend to share common features indicating recent changes, presumably in response to specific selective pressures in each region. Analysis of Ax270 and Ex003 showed that, along with other antibiotic-susceptible clinical strains (AB307-0294 and MRSN7213, both recovered in the USA), they represent additional lineages within the GC1 population. This suggests the presence of presumably antibiotic-susceptible GC1 lineages in the environment and that the real GC1 population is more diverse than currently perceived. These lineages can evolve under antibiotic selective pressure by acquiring resistance determinants, and in turn become potentially problematic. However, additional antibiotic-susceptible strains from more diverse geographical regions would be needed to better draw the population structure of GC1 and all its sublineages.

## Supplementary Data

Supplementary material 1
