# Supplementary material for: Phylogenomics of two ST1 antibiotic-susceptible non-clinical Acinetobacter baumannii strains reveals multiple lineages and complex evolutionary history in global clone 1
Source: Microb Genom. 2021 Dec 7;7(12):000705. doi: 10.1099/mgen.0.000705 (PMC8767349; doi:10.1099/mgen.0.000705)
Supplement: Supplementary material 1 [file mgen-7-0705-s001.pdf]

```
<comM left segment Insert 5′-end>
TTCTATTTTTAGATGAACCTCATATTTCCCTAAATAAGTTAAGTGTCATTTAATCTTTA
     || || | | |       | ||||| |  |      |  |    |  |  | |
ACAACTTATTTGCTAATTAGGGAAATTCCCGAGTTTGACAGAAAGGTTCTAGAAGTCTT
           <Insert 3′-end comM right segment>
```

Fig. S1. Alignment of the boundaries of the *comM* insert in Ex003. Letters coloured blue indicate the sequence of *comM* and those shown in dark gold represents the insert. Identical sequences are shown using vertical bars.

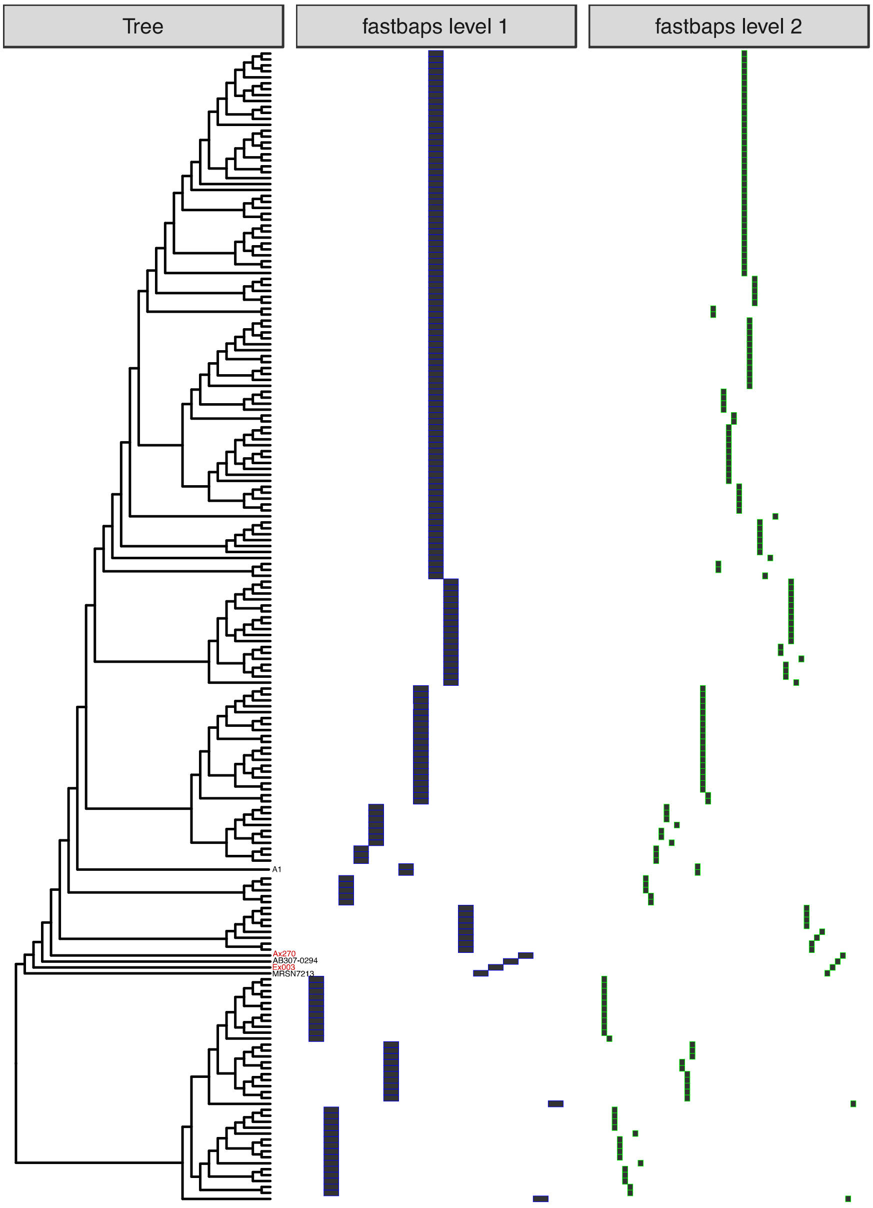

Fig. S2. BAPS phylogenetic groups drawn using the fastbaps method. Vertical lines indicate level 1 and 2 BAPS groups.

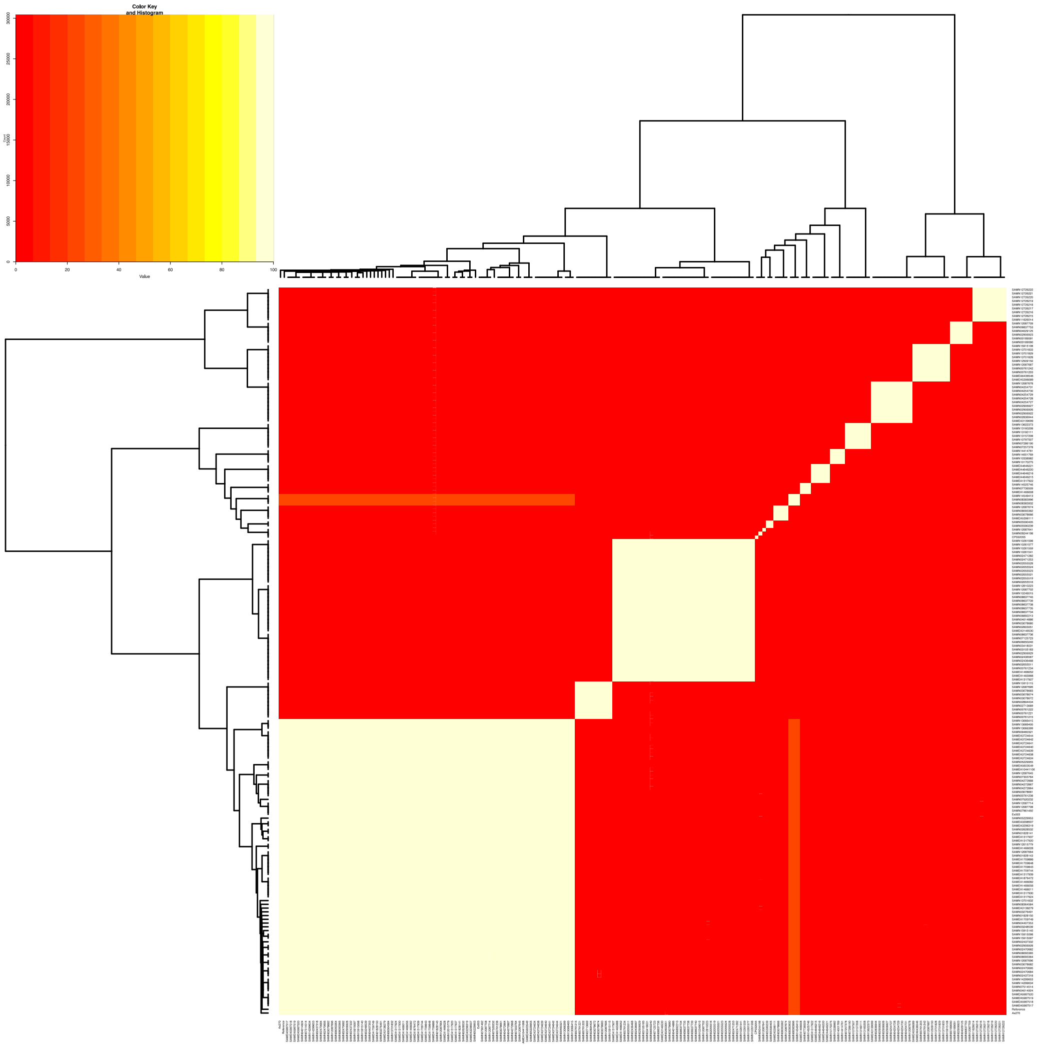

Fig. S3. Heatmaps illustrating the stability of the inferred clusters using the Bootstrap

Table S1. Antibiotic resistance profiles of Ax270 and Ex003†

Antibiotic/ Strain	Ax270	Ex003
Ampicillin	6	6
Ampicillin/Sulbactam	12	10
Ceftriaxone	6	5
Ceftazidime	8	7
Cefotaxime	7	6
Imipenem	13	13
Meropenem	12	11
Netilmicin	10	8
Amikacin	9	8
Gentamicin	9	9
Tobramycin	10	8
Kanamycin	10	9
Neomycin	9	7
Streptomycin	7	6
Spectinomycin	5	5
Sulfamethoxazole	10	10
Trimethoprim	4	4
Rifampicin	6	6
Nalidixic acid	0	7
Ciprofloxacin	7	9
Tetracycline	7	7
Chloramphenicol	0	0

† numbers indicate the annular radius of growth inhibition zones in millimetres.

Strain	ST	Location	IsolationSource	IsolationType	CollectionDate	BioSample_prok	Assembly_prok	Level_prok	Size_Mb_	GC_	Replicons	WGS	Scaffolds	No. of Contigs	N50	Antibiotic resistance genes
ACB5	1	Bosnia and Herzegovina	urine	clinical	8/4/11	SAMEA104411068	GCA_900240205.1	Contig	3.89807	38.9		OEON01	118	118	211235	aac(3)-Ia=COMPLETE,aadA1=COMPLETE,abaF=COMPLETE,adeC=COMPLETE,amvA=COMPLETE,ant(3'')-IIa=PARTIAL,blaADC-74=COMPLETE,blaOXA-69=COMPLETE,blaOXA-72=COMPLETE,sul1=COMPLETE
D81	1	Australia: Sydney	wound	clinical	2010	SAMEA1317920	GCA_001511995.1	Scaffold	4.01111	39		FBXC01	106	120	163775	aac(3)-Ia=COMPLETE,aadA1=COMPLETE,abaF=COMPLETE,adeC=COMPLETE,amvA=COMPLETE,ant(3'')-IIa=COMPLETE,aph(3')-VIa=COMPLETE,blaADC-11=COMPLETE,blaOXA-58=COMPLETE,blaOXA-69=COMPLETE,catA1=COMPLETE,sul1=COMPLETE,tet(A)=COMPLETE
D13	1	Australia: Sydney	urine	clinical	2009	SAMEA1317922	GCA_001512155.1	Scaffold	4.04917	39.1		FBXI01	112	146	151483	aac(3)-Ia=COMPLETE,aadA1=COMPLETE,abaF=COMPLETE,adeC=COMPLETE,amvA=COMPLETE,ant(3'')-IIa=COMPLETE,aph(3')-Ia=COMPLETE,blaADC-176=COMPLETE,blaADC=COMPLETE,blaOXA-69=COMPLETE,sul1=COMPLETE
D2	1	Australia: Sydney	wound	clinical	2006	SAMEA1317924	GCA_001512035.1	Scaffold	3.95678	39		FBWY01	96	107	170867	aac(3)-Ia=COMPLETE,aadA1=COMPLETE,abaF=COMPLETE,adeC=COMPLETE,amvA=COMPLETE,ant(3'')-IIa=COMPLETE,aph(3')-Ia=COMPLETE,blaADC-11=COMPLETE,blaOXA-69=COMPLETE,sul1=COMPLETE
RBH3	1	Australia: Brisbane	endotracheal aspirate	clinical	2002	SAMEA1317927	GCA_001512075.1	Scaffold	4.0114	39.1		FBXD01	132	145	185929	aac(3)-Ia=COMPLETE,aadA1=COMPLETE,abaF=COMPLETE,adeC=COMPLETE,amvA=COMPLETE,ant(3'')-IIa=COMPLETE,aph(3')-Ia=COMPLETE,blaADC-176=COMPLETE,blaADC=COMPLETE,blaOXA-69=COMPLETE,blaTEM-1=COMPLETE,catA1=COMPLETE,sul1=COMPLETE,tet(A)=COMPLETE
D3208	1	Australia: Sydney	blood	clinical	1997	SAMEA1317930	GCA_001512255.1	Scaffold	3.94043	39.1		FBWZ01	89	97	272409	aac(3)-Ia=COMPLETE,aadA1=COMPLETE,abaF=COMPLETE,adeC=COMPLETE,amvA=COMPLETE,ant(3'')-IIa=COMPLETE,aph(3')-Ia=COMPLETE,blaADC-11=COMPLETE,blaOXA-69=COMPLETE,sul1=COMPLETE,tet(A)=COMPLETE
D78	1	Australia: Sydney		clinical	2010	SAMEA1317937	GCA_001511915.1	Scaffold	4.01032	39		FBXH01	105	116	163775	aac(3)-Ia=COMPLETE,aadA1=COMPLETE,abaF=COMPLETE,adeC=COMPLETE,amvA=COMPLETE,ant(3'')-IIa=COMPLETE,aph(3')-VIa=COMPLETE,blaADC-11=COMPLETE,blaOXA-58=COMPLETE,blaOXA-69=COMPLETE,catA1=COMPLETE,sul1=COMPLETE,tet(A)=COMPLETE
WM98	1	Australia: Sydney	wound	clinical	1998	SAMEA1317939	GCA_001512015.1	Scaffold	4.06916	39		FBXB01	81	95	222934	aac(3)-Ia=COMPLETE,aadA1=COMPLETE,abaF=COMPLETE,adeC=COMPLETE,amvA=COMPLETE,ant(3'')-IIa=COMPLETE,aph(3')-Ia=COMPLETE,blaADC-11=COMPLETE,blaOXA-69=COMPLETE,blaTEM-1=COMPLETE,catA1=COMPLETE,sul1=COMPLETE,tet(A)=COMPLETE
6772166	1	Australia: Adelaide	pus	clinical	2002	SAMEA1465988	GCA_001512175.1	Scaffold	4.00129	39.1		FBWX01	118	132	171004	aac(3)-Ia=COMPLETE,aadA1=COMPLETE,abaF=COMPLETE,adeC=COMPLETE,amvA=COMPLETE,ant(3'')-IIa=COMPLETE,aph(3')-Ia=COMPLETE,blaADC-11=COMPLETE,blaADC=COMPLETE,blaOXA-69=COMPLETE,blaTEM-1=COMPLETE,catA1=COMPLETE,sul1=COMPLETE,tet(A)=COMPLETE
A92	1	Australia: Sydney	urine	clinical	2005	SAMEA1466011	GCA_001511975.1	Scaffold	3.90006	39		FBWV01	57	72	259179	aac(3)-Ia=COMPLETE,aadA1=COMPLETE,abaF=COMPLETE,adeC=COMPLETE,amvA=COMPLETE,ant(3'')-IIa=COMPLETE,aph(3')-Ia=COMPLETE,blaADC-11=COMPLETE,blaOXA-69=COMPLETE,sul1=COMPLETE
D30	1	Australia: Sydney	wound	clinical	2008	SAMEA1466056	GCA_001511935.1	Scaffold	3.88057	39		FBXG01	65	73	277358	aac(3)-Ia=COMPLETE,aadA1=COMPLETE,abaF=COMPLETE,adeC=COMPLETE,amvA=COMPLETE,ant(3'')-IIa=COMPLETE,aph(3')-Ia=COMPLETE,blaADC-11=COMPLETE,blaOXA-69=COMPLETE,sul1=COMPLETE
A83	1	Australia: Sydney		clinical	2002	SAMEA1466082	GCA_001512235.1	Scaffold	3.85864	39		FBWU01	53	64	272130	aac(3)-Ia=COMPLETE,aadA1=COMPLETE,abaF=COMPLETE,adeC=COMPLETE,amvA=COMPLETE,ant(3'')-IIa=COMPLETE,aph(3')-Ia=COMPLETE,blaADC-11=COMPLETE,blaOXA-69=COMPLETE,sul1=COMPLETE
J1	1	Australia: Sydney		clinical	1995	SAMEA1709744	GCA_001511875.1	Scaffold	3.964	39.1		FBWQ01	59	65	374056	aac(3)-Ia=COMPLETE,aadA1=COMPLETE,abaF=COMPLETE,adeC=COMPLETE,amvA=COMPLETE,ant(3'')-IIa=COMPLETE,aph(3')-Ia=COMPLETE,blaADC-11=COMPLETE,blaOXA-69=COMPLETE,blaTEM-1=COMPLETE,catA1=COMPLETE,sul1=COMPLETE,tet(A)=COMPLETE
A297	1	Netherlands		clinical	1984	SAMEA1709749	GCA_001512215.1	Scaffold	4.10978	39.1		FBWR01	92	99	284036	abaF=COMPLETE,adeC=COMPLETE,amvA=COMPLETE,ant(2'')-Ia=COMPLETE,ant(3'')-IIa=COMPLETE,aph(3'')-Ib=COMPLETE,aph(3')-Ia=COMPLETE,aph(6)-Id=COMPLETE,blaADC-11=COMPLETE,blaOXA-69=COMPLETE,blaTEM-1=COMPLETE,catA1=COMPLETE,dfrA5=COMPLETE,sul1=COMPLETE,sul2=COMPLETE,tet(A)=COMPLETE
J10	1	Australia: Sydney		clinical	1999	SAMEA1709843	GCA_001511855.1	Scaffold	4.07907	39		FBWS01	70	84	222245	aac(3)-Ia=COMPLETE,aadA1=COMPLETE,abaF=COMPLETE,adeC=COMPLETE,amvA=COMPLETE,ant(3'')-IIa=COMPLETE,aph(3')-Ia=COMPLETE,blaADC-11=COMPLETE,blaOXA-69=COMPLETE,blaTEM-1=COMPLETE,catA1=COMPLETE,sul1=COMPLETE,tet(A)=COMPLETE
J7	1	Australia: Sydney		clinical	1998	SAMEA1709848	GCA_001512115.1	Scaffold	4.06953	39		FBWT01	65	75	345806	aac(3)-Ia=COMPLETE,aadA1=COMPLETE,abaF=COMPLETE,adeC=COMPLETE,amvA=COMPLETE,ant(3'')-IIa=COMPLETE,aph(3')-Ia=COMPLETE,blaADC-11=COMPLETE,blaOXA-69=COMPLETE,blaTEM-1=COMPLETE,catA1=COMPLETE,sul1=COMPLETE,tet(A)=COMPLETE
J5	1	Australia: Sydney		clinical	1997	SAMEA1709886	GCA_001511895.1	Scaffold	3.96801	39.1		FBWP01	58	68	293405	aac(3)-Ia=COMPLETE,aadA1=COMPLETE,abaF=COMPLETE,adeC=COMPLETE,amvA=COMPLETE,ant(3'')-IIa=COMPLETE,aph(3')-Ia=COMPLETE,blaADC-11=COMPLETE,blaOXA-69=COMPLETE,blaTEM-1=COMPLETE,catA1=COMPLETE,sul1=COMPLETE,tet(A)=COMPLETE
Ab118	1	Australia		clinical	2008	SAMEA1876472	GCA_900490095.1	Scaffold	3.94726	39		UEJD01	61	74	199788	aac(3)-I=COMPLETE,aadA1=COMPLETE,adeC=COMPLETE,amvA=COMPLETE,ant(3'')-IIa=COMPLETE,aph(3')-Ia=COMPLETE,blaADC-11=COMPLETE,blaOXA-69=COMPLETE,sul1=COMPLETE
AYE	1	France		environmental/other		SAMEA3138279	GCA_000069245.1	Complete	4.04873	39.3357	chromosome:NC_010410.		5	5	3936291	aac(3)-Ia=COMPLETE,aacA57-2=COMPLETE,aadA1=COMPLETE,abaF=COMPLETE,adeC=COMPLETE,amvA=COMPLETE,ant(2'')-Ia=COMPLETE,ant(3'')-IIa=COMPLETE,aph(3'')-Ib=COMPLETE,aph(3')-Ia=COMPLETE,aph(6)-Id=COMPLETE,arr-2=COMPLETE,blaADC-11=COMPLETE,blaOXA-10=COMPLETE,blaOXA-69=COMPLETE,blaVEB-1=COMPLETE,catA1=COMPLETE,cmlA5=C
Pulsotype 27	94	United Kingdom		environmental/other	9/2/11	SAMEA3139099	GCA_000750855.1	Contig	4.03141	38.8		CBXD01	160	160	77550	abaF=COMPLETE,adeC=COMPLETE,amvA=COMPLETE,ant(2'')-Ia=COMPLETE,ant(3'')-IIa=COMPLETE,aph(3')-Ia=COMPLETE,aph(3')-VIa=COMPLETE,blaADC=COMPLETE,blaOXA-23=COMPLETE,blaOXA-69=COMPLETE,mph(E)=COMPLETE,msr(E)=COMPLETE,sul2=COMPLETE
NCTC13421	1	United Kingdom	not available: to be re	clinical	2004	SAMEA3146530	GCA_900478145.1	Complete	4.04416	39.2	chromosome 1:NZ_LS483		1	1	4044162	aac(3)-Ia=COMPLETE,aadA1=COMPLETE,abaF=COMPLETE,adeC=COMPLETE,amvA=COMPLETE,ant(3'')-IIa=COMPLETE,aph(3')-Ia=COMPLETE,blaADC-11=COMPLETE,blaADC-176=COMPLETE,blaOXA-23=COMPLETE,blaOXA-69=COMPLETE,blaTEM-1=COMPLETE,catA1=COMPLETE,sul1=COMPLETE,tet(A)=COMPLETE
CHI-45-1	1	India	Clinical sample, endo	environmental/other		SAMEA3296319	GCA_001373535.1	Scaffold	3.94559	39.3		CVLE01	165	192	101555	aac(3)-IId=COMPLETE,aac(3)-Ia=COMPLETE,aadA1=COMPLETE,aadA2=COMPLETE,abaF=COMPLETE,adeC=COMPLETE,amvA=COMPLETE,ant(3'')-IIa=COMPLETE,aph(3')-Ia=COMPLETE,armA=COMPLETE,blaADC-78=COMPLETE,blaNDM-1=COMPLETE,blaOXA-23=COMPLETE,blaOXA-69=COMPLETE,blaTEM-1=COMPLETE,ble=COMPLETE,catA1=COMPLETE,dfrA12=COMPLETI
CHI-34	1	India	Clinical sample, sputu	environmental/other		SAMEA3298507	GCA_001373575.1	Scaffold	4.03901	39.1		CVLD01	140	163	132163	aac(3)-IId=COMPLETE,aac(3)-Ia=COMPLETE,aadA1=COMPLETE,abaF=COMPLETE,adeC=COMPLETE,amvA=COMPLETE,ant(3'')-IIa=COMPLETE,aph(3')-Ia=COMPLETE,blaADC-78=COMPLETE,blaNDM-1=COMPLETE,blaOXA-23=COMPLETE,blaOXA-69=COMPLETE,blaTEM-1=COMPLETE,ble=COMPLETE,catA1=COMPLETE,floR=COMPLETE,sul1=COMPLETE,tet(A)=COMPLETE
B	1	Germany	tracheal secretion	clinical	8/13/08	SAMEA3724634	GCA_900020545.1	Contig	3.98936	38.9		FITR01	212	212	66983	aac(3)-Ia=COMPLETE,aadA1=COMPLETE,abaF=COMPLETE,adeC=COMPLETE,amvA=COMPLETE,ant(3'')-IIa=COMPLETE,aph(3')-VIa=COMPLETE,blaADC-186=COMPLETE,blaOXA-164=COMPLETE,blaOXA-69=COMPLETE,sul1=COMPLETE
F	1	Germany	tracheostoma	clinical	8/20/08	SAMEA3724638	GCA_900029355.1	Contig	3.93849	38.9		FCNJ01	152	152	110179	aac(3)-Ia=COMPLETE,aadA1=COMPLETE,abaF=COMPLETE,adeC=COMPLETE,amvA=COMPLETE,ant(3'')-IIa=COMPLETE,aph(3')-VIa=COMPLETE,blaADC-186=COMPLETE,blaOXA-164=COMPLETE,blaOXA-69=COMPLETE,sul1=COMPLETE
G	1	Germany	tracheal secretion	clinical	8/26/08	SAMEA3724639	GCA_900042755.1	Contig	3.98613	38.9		FCNF01	223	223	52313	aac(3)-Ia=COMPLETE,aadA1=COMPLETE,abaF=COMPLETE,adeC=COMPLETE,amvA=COMPLETE,ant(3'')-IIa=COMPLETE,aph(3')-VIa=COMPLETE,blaADC-186=COMPLETE,blaOXA-58=COMPLETE,blaOXA-69=COMPLETE,sul1=COMPLETE
I	1	Germany	tracheal secretion	clinical	8/29/08	SAMEA3724640	GCA_900029375.1	Contig	3.98481	38.9		FCNK01	164	164	70284	aac(3)-Ia=COMPLETE,aadA1=COMPLETE,abaF=COMPLETE,adeC=COMPLETE,amvA=COMPLETE,ant(3'')-IIa=COMPLETE,aph(3')-VIa=COMPLETE,blaADC-186=COMPLETE,blaOXA-58=COMPLETE,blaOXA-69=COMPLETE,sul1=COMPLETE
J	1	Germany	bedsore	clinical	9/24/08	SAMEA3724641	GCA_900043985.1	Contig	3.98118	38.9		FCNH01	122	122	109052	aac(3)-Ia=COMPLETE,aadA1=COMPLETE,abaF=COMPLETE,adeC=COMPLETE,amvA=COMPLETE,ant(3'')-IIa=COMPLETE,aph(3')-VIa=COMPLETE,blaADC-186=COMPLETE,blaOXA-58=COMPLETE,blaOXA-69=COMPLETE,sul1=COMPLETE
K	1	Germany	blood culture	clinical	10/14/08	SAMEA3724642	GCA_900043995.1	Contig	3.97861	38.9		FCNI01	183	183	69102	aac(3)-Ia=COMPLETE,aadA1=COMPLETE,abaF=COMPLETE,adeC=COMPLETE,amvA=COMPLETE,ant(3'')-IIa=COMPLETE,aph(3')-VIa=COMPLETE,blaADC-186=COMPLETE,blaOXA-58=COMPLETE,blaOXA-69=COMPLETE,sul1=COMPLETE
M	1	Germany	tracheal secretion	clinical	10/16/08	SAMEA3724644	GCA_900067075.1	Contig	3.98079	38.9		FJMY01	161	161	61197	aac(3)-Ia=COMPLETE,aadA1=COMPLETE,abaF=COMPLETE,adeC=COMPLETE,amvA=COMPLETE,ant(3'')-IIa=COMPLETE,aph(3')-VIa=COMPLETE,blaADC-186=COMPLETE,blaOXA-58=COMPLETE,blaOXA-69=COMPLETE,sul1=COMPLETE
KCRI-363	1	Tanzania	wound swab sample	clinical	12/4/14	SAMEA4646215	GCA_900406715.1	Contig	4.14198	39.2		OVCI01	165	165	229289	aac(3)-Ia=COMPLETE,aadA1=COMPLETE,abaF=COMPLETE,adeC=COMPLETE,amvA=COMPLETE,ant(3'')-IIa=COMPLETE,aph(3')-Ia=COMPLETE,blaADC=COMPLETE,blaOXA-69=COMPLETE,catA1=COMPLETE,sul1=COMPLETE,tet(A)=COMPLETE
KCRI-423	1	Tanzania	wound swab sample	clinical	3/4/15	SAMEA4646216	GCA_900406745.1	Contig	4.07983	39.2		OVCG01	184	184	447289	aac(3)-Ia=COMPLETE,aadA1=COMPLETE,abaF=COMPLETE,adeC=COMPLETE,amvA=COMPLETE,ant(3'')-IIa=COMPLETE,aph(3')-Ia=COMPLETE,blaADC=COMPLETE,blaOXA-69=COMPLETE,catA1=COMPLETE,sul1=COMPLETE,tet(A)=COMPLETE
KCRI-518B	1	Tanzania	wound swab sample	clinical	5/30/15	SAMEA4646220	GCA_900406765.1	Contig	4.14835	39		OVCL01	166	166	309557	aac(3)-Ia=COMPLETE,aadA1=COMPLETE,abaF=COMPLETE,adeC=COMPLETE,amvA=COMPLETE,ant(3'')-IIa=COMPLETE,aph(3')-Ia=COMPLETE,blaADC=COMPLETE,blaOXA-69=COMPLETE,catA1=COMPLETE,sul1=COMPLETE,tet(A)=COMPLETE
KCRI-558	1	Tanzania	wound swab sample	clinical	7/14/15	SAMEA4646221	GCA_900406755.1	Contig	4.07939	39.1		OVCM01	125	125	440462	aac(3)-Ia=COMPLETE,aadA1=COMPLETE,abaF=COMPLETE,adeC=COMPLETE,amvA=COMPLETE,ant(3'')-IIa=COMPLETE,aph(3')-Ia=COMPLETE,blaADC=COMPLETE,blaOXA-23=COMPLETE,blaOXA-69=COMPLETE,catA1=COMPLETE,sul1=COMPLETE,tet(A)=COMPLETE
Aci00834	315	Germany: Heidelberg	clinical material	clinical	5/28/13	SAMEA5396089	GCA_005819735.1	Contig	3.92447	39		VAFS01	135	135	65643	abaF=COMPLETE,adeC=COMPLETE,amvA=COMPLETE,ant(2'')-Ia=PARTIAL_END_OF_CONTIG,ant(3'')-IIa=COMPLETE,blaADC-79=COMPLETE,blaOXA-23=COMPLETE,blaOXA-69=COMPLETE,catA1=COMPLETE,sul2=COMPLETE,tet(B)=COMPLETE
Aci00872	717	Germany: Heidelberg	clinical material	clinical	9/12/15	SAMEA5396111	GCA_005819215.1	Contig	4.07159	38.9		VAGO01	134	134	83421	abaF=COMPLETE,adeC=COMPLETE,amvA=COMPLETE,ant(2'')-Ia=PARTIAL_END_OF_CONTIG,ant(3'')-IIa=COMPLETE,aph(3')-VIa=COMPLETE,aph(3')-VIa=PARTIAL_END_OF_CONTIG,blaADC=COMPLETE,blaOXA-23=COMPLETE,blaOXA-69=COMPLETE,blaOXA=PARTIAL_END_OF_CONTIG,sul2=COMPLETE
aba_5m	1			environmental/other		SAMEA5603549	GCA_901484875.1	Contig	3.89807	38.9		CABEFJ01	118	118	211235	aac(3)-Ia=COMPLETE,aadA1=COMPLETE,abaF=COMPLETE,adeC=COMPLETE,amvA=COMPLETE,ant(3'')-IIa=PARTIAL,blaADC-74=COMPLETE,blaOXA-69=COMPLETE,blaOXA-72=COMPLETE,sul1=COMPLETE
Aci00879	1	South Africa	clinical material	clinical	2017	SAMEA5987517	GCA_902702975.1	Contig	4.0567	39.2		CACRXQ01	92	92	128723	aac(3)-Ia=COMPLETE,aadA1=PARTIAL_END_OF_CONTIG,abaF=COMPLETE,adeC=COMPLETE,amvA=COMPLETE,ant(3'')-IIa=COMPLETE,aph(3'')-Ib=COMPLETE,aph(3')-Ia=COMPLETE,aph(6)-Id=PARTIAL_END_OF_CONTIG,armA=COMPLETE,arr-2=COMPLETE,blaADC=COMPLETE,blaNDM-1=COMPLETE,blaOXA-23=COMPLETE,blaOXA-69=COMPLETE,blaPER-7=COMPLETE,bl
Aci00880	1	South Africa	clinical material	clinical	2017	SAMEA5987518	GCA_902703085.1	Contig	4.0195	39.2		CACRYG01	87	87	125053	aac(3)-Ia=COMPLETE,aadA1=PARTIAL_END_OF_CONTIG,abaF=COMPLETE,adeC=COMPLETE,amvA=COMPLETE,ant(3'')-IIa=COMPLETE,aph(3'')-Ib=COMPLETE,aph(3')-Ia=COMPLETE,aph(6)-Id=PARTIAL_END_OF_CONTIG,armA=COMPLETE,arr-2=COMPLETE,blaADC=COMPLETE,blaNDM-1=COMPLETE,blaOXA-23=COMPLETE,blaOXA-69=COMPLETE,blaPER-7=COMPLETE,bl
Aci00881	1	South Africa	clinical material	clinical	2017	SAMEA5987519	GCA_902703065.1	Contig	4.05857	39.2		CACRXX01	90	90	134272	aac(3)-Ia=COMPLETE,aadA1=PARTIAL_END_OF_CONTIG,abaF=COMPLETE,adeC=COMPLETE,amvA=COMPLETE,ant(3'')-IIa=COMPLETE,aph(3'')-Ib=COMPLETE,aph(3')-Ia=COMPLETE,aph(6)-Id=PARTIAL_END_OF_CONTIG,armA=COMPLETE,arr-2=COMPLETE,blaADC=COMPLETE,blaNDM-1=COMPLETE,blaOXA-23=COMPLETE,blaOXA-69=COMPLETE,blaPER-7=COMPLETE,bl
Aci00882	1	South Africa	clinical material	clinical	2017	SAMEA5987520	GCA_902703115.1	Contig	4.05855	39.2		CACRXT01	89	89	160646	aac(3)-Ia=COMPLETE,aadA1=PARTIAL_END_OF_CONTIG,abaF=COMPLETE,adeC=COMPLETE,amvA=COMPLETE,ant(3'')-IIa=COMPLETE,aph(3'')-Ib=COMPLETE,aph(3')-Ia=COMPLETE,aph(6)-Id=PARTIAL_END_OF_CONTIG,armA=COMPLETE,arr-2=COMPLETE,blaADC=COMPLETE,blaNDM-1=COMPLETE,blaOXA-23=COMPLETE,blaOXA-69=COMPLETE,blaPER-7=COMPLETE,bl
AC3-M	315	Morocco		clinical	4/13/15	SAMEA6436546	GCA_902713735.1	Contig	3.90974	39.1		CACTJA01	193	193	44697	abaF=COMPLETE,adeC=COMPLETE,amvA=COMPLETE,ant(3'')-IIa=COMPLETE,aph(3')-Ia=COMPLETE,aph(3')-VIa=COMPLETE,blaADC=PARTIAL_END_OF_CONTIG,blaOXA-23=COMPLETE,blaOXA-69=COMPLETE,catA1=COMPLETE,sul2=COMPLETE,tet(B)=COMPLETE
6013113	81	not determined	skin	clinical		SAMN00189090	GCA_000163375.2	Scaffold	3.99904	38.9		ACYR02	118	230	39772	abaF=COMPLETE,adeC=COMPLETE,amvA=COMPLETE,ant(2'')-Ia=COMPLETE,ant(3'')-IIa=COMPLETE,aph(3')-Ia=COMPLETE,blaADC=COMPLETE,blaOXA-69=COMPLETE,sul2=COMPLETE
6013150	81	not determined	skin	clinical		SAMN00189091	GCA_000163355.2	Scaffold	3.98724	38.9		ACYQ02	202	214	47274	abaF=COMPLETE,adeC=COMPLETE,amvA=COMPLETE,ant(2'')-Ia=COMPLETE,ant(3'')-IIa=COMPLETE,aph(3')-Ia=COMPLETE,blaADC=COMPLETE,blaOXA-69=COMPLETE,sul2=COMPLETE
OIFC074	19			environmental/other		SAMN00761203	GCA_000305215.1	Contig	3.93589	39.1		AMDE01	66	66	139991	adeC=COMPLETE,amvA=COMPLETE,ant(3'')-IIa=COMPLETE,blaADC-79=COMPLETE,blaOXA-69=COMPLETE,catA1=COMPLETE,sul2=COMPLETE,tet(B)=COMPLETE
IS-58	1			environmental/other		SAMN00761215	GCA_000297595.1	Contig	4.06389	39		AMGH01	61	61	276191	aac(6')-Ib3=COMPLETE,aadA2=COMPLETE,abaF=COMPLETE,adeC=COMPLETE,amvA=COMPLETE,ant(2'')-Ia=COMPLETE,ant(3'')-IIa=COMPLETE,aph(3'')-Ib=COMPLETE,aph(3')-VIa=COMPLETE,aph(6)-Id=COMPLETE,blaADC-11=COMPLETE,blaGES-11=COMPLETE,blaOXA-23=COMPLETE,blaOXA-69=COMPLETE,cmlA1=COMPLETE,dfrA7=COMPLETE,sul1=COMPLETE
IS-235	1			environmental/other		SAMN00761221	GCA_000304695.1	Contig	4.06039	38.9		AMEI01	76	76	151506	aac(6')-Ib3=COMPLETE,aadA2=COMPLETE,abaF=COMPLETE,adeC=COMPLETE,amvA=COMPLETE,ant(2'')-Ia=COMPLETE,ant(3'')-IIa=COMPLETE,aph(3'')-Ib=COMPLETE,aph(3')-VIa=COMPLETE,aph(6)-Id=COMPLETE,blaADC-11=COMPLETE,blaGES-11=COMPLETE,blaOXA-23=COMPLETE,blaOXA-69=COMPLETE,cmlA1=COMPLETE,dfrA7=COMPLETE,sul1=COMPLETE
IS-251	1			environmental/other		SAMN00761222	GCA_000304655.1	Contig	4.00729	38.9		AMEJ01	72	72	244064	aac(6')-Ib3=COMPLETE,aadA2=COMPLETE,abaF=COMPLETE,adeC=COMPLETE,amvA=COMPLETE,ant(2'')-Ia=COMPLETE,ant(3'')-IIa=COMPLETE,aph(3'')-Ib=COMPLETE,aph(3')-VIa=COMPLETE,aph(6)-Id=COMPLETE,blaADC-11=COMPLETE,blaGES-11=COMPLETE,blaOXA-23=COMPLETE,blaOXA-69=COMPLETE,cmlA1=COMPLETE,dfrA7=COMPLETE,sul1=COMPLETE
Canada BC1	1			environmental/other		SAMN00761234	GCA_000309275.1	Contig	3.9364	39.1		AMSZ01	66	66	190509	aac(3)-Ia=COMPLETE,aadA1=COMPLETE,abaF=COMPLETE,adeC=COMPLETE,amvA=COMPLETE,ant(3'')-IIa=COMPLETE,blaOXA-23=COMPLETE,blaOXA-69=COMPLETE,catA1=COMPLETE,sul1=PARTIAL,tet(A)=COMPLETE
Naval-83	20			environmental/other		SAMN00761238	GCA_000305315.1	Contig	4.1066	39		AMFK01	103	103	138386	abaF=COMPLETE,adeC=COMPLETE,amvA=COMPLETE,ant(3'')-IIa=COMPLETE,aph(3')-VIa=COMPLETE,blaADC-74=COMPLETE,blaOXA-23=COMPLETE,blaOXA-69=COMPLETE,sul1=COMPLETE
Naval-21	19			environmental/other		SAMN00761242	GCA_000309195.1	Contig	3.9238	38.9		AMSY01	75	75	94859	adeC=COMPLETE,amvA=COMPLETE,ant(3'')-IIa=COMPLETE,aph(3')-Ia=COMPLETE,blaADC-79=COMPLETE,blaOXA-69=COMPLETE,sul2=COMPLETE,tet(B)=COMPLETE
ANC 4097	1	Czech Republic: Usti nad l	tracheal aspirate	clinical	2011	SAMN01828141	GCA_000369365.1	Scaffold	4.25258	39.1		APRF01	19	31	418250	aac(3)-Ia=COMPLETE,aadA1=COMPLETE,abaF=COMPLETE,adeC=COMPLETE,amvA=COMPLETE,ant(3'')-IIa=COMPLETE,aph(3')-Ia=COMPLETE,blaADC-11=COMPLETE,blaNDM-1=COMPLETE,blaOXA-23=COMPLETE,blaOXA-69=COMPLETE,blaTEM-1=COMPLETE,ble=COMPLETE,catA1=COMPLETE,sul1=PARTIAL,sul1=PARTIAL_END_OF_CONTIG,tet(A)=COMPLETE
NIPH 290	1	Czech Republic: Pribram	urine	clinical	1994	SAMN01828143	GCA_000369325.1	Scaffold	4.02649	39.3		APRD01	14	28	391506	aac(3)-Ia=COMPLETE,aadA1=COMPLETE,adeC=COMPLETE,amvA=COMPLETE,ant(3'')-IIa=COMPLETE,aph(3')-VIa=COMPLETE,blaADC-11=COMPLETE,blaOXA-69=COMPLETE,blaTEM-1=COMPLETE,catA1=COMPLETE,sul1=COMPLETE,tet(A)=COMPLETE
NIPH 527	1	Netherlands: Dordrecht	urine	clinical	1984	SAMN01828150	GCA_000369185.1	Scaffold	4.188	39.2		APQW01	18	31	412856	abaF=COMPLETE,adeC=COMPLETE,amvA=COMPLETE,ant(2'')-Ia=COMPLETE,ant(3'')-IIa=COMPLETE,aph(3'')-Ib=COMPLETE,aph(3')-Ia=COMPLETE,aph(6)-Id=COMPLETE,blaADC-11=COMPLETE,blaOXA-69=COMPLETE,blaTEM-1=COMPLETE,catA1=COMPLETE,dfrA5=COMPLETE,sul1=PARTIAL,sul2=COMPLETE,tet(A)=COMPLETE
TG20277	1	Germany	sputum	clinical	2006	SAMN02055511	GCA_000453885.1	Contig	4.1105	39.2		ASFH01	62	62	205560	aac(3)-Ia=COMPLETE,aadA1=COMPLETE,abaF=COMPLETE,adeC=COMPLETE,amvA=COMPLETE,ant(3'')-IIa=COMPLETE,aph(3')-Ia=COMPLETE,blaADC-176=COMPLETE,blaADC=PARTIAL_END_OF_CONTIG,blaOXA-23=COMPLETE,blaOXA-69=COMPLETE,blaTEM-1=COMPLETE,catA1=COMPLETE,sul1=COMPLETE,tet(A)=COMPLETE
TG22112	1	USA	tracheal aspirate	clinical	2011	SAMN02055516	GCA_000453945.1	Contig	4.38026	38.9		ASFK01	64	64	176021	abaF=COMPLETE,adeC=COMPLETE,amvA=COMPLETE,ant(3'')-IIa=COMPLETE,armA=COMPLETE,blaADC=PARTIAL_END_OF_CONTIG,blaOXA-23=COMPLETE,blaOXA-69=COMPLETE,mph(E)=COMPLETE,msr(E)=COMPLETE,sul1=COMPLETE
TG22148	1	USA	tracheal aspirate	clinical	2011	SAMN02055519	GCA_000454005.1	Contig	4.31569	38.9		ASFN01	54	54	182896	abaF=COMPLETE,adeC=COMPLETE,amvA=COMPLETE,ant(3'')-IIa=COMPLETE,aph(3')-Ia=COMPLETE,armA=COMPLETE,blaADC-11=COMPLETE,blaOXA-23=COMPLETE,blaOXA-69=COMPLETE,mph(E)=COMPLETE,msr(E)=COMPLETE,sul1=COMPLETE
TG22190	1	USA	tracheal aspirate	clinical	2011	SAMN02055521	GCA_000453045.1	Contig	4.12273	38.9		ASFP01	48	48	205363	abaF=COMPLETE,adeC=COMPLETE,amvA=COMPLETE,ant(3'')-IIa=COMPLETE,blaADC-11=COMPLETE,blaADC-176=COMPLETE,blaOXA-23=COMPLETE,blaOXA-69=COMPLETE
TG22194	1	USA	tracheal aspirate	clinical	2009	SAMN02055523	GCA_000453085.1	Contig	4.13536	39		ASFR01	61	61	203397	aac(6')-Ib=COMPLETE,aadA1=COMPLETE,abaF=COMPLETE,adeC=COMPLETE,amvA=COMPLETE,ant(3'')-IIa=COMPLETE,aph(3')-Ia=COMPLETE,blaADC=PARTIAL_END_OF_CONTIG,blaOXA-23=COMPLETE,blaOXA-69=COMPLETE,catB8=COMPLETE,mph(E)=COMPLETE,msr(E)=COMPLETE,sul1=COMPLETE
TG22196	1	USA	blood	clinical	2009	SAMN02055524	GCA_000453105.1	Contig	3.99864	39		ASFS01	43	43	279804	abaF=COMPLETE,adeC=COMPLETE,amvA=COMPLETE,ant(3'')-IIa=COMPLETE,aph(3')-Ia=COMPLETE,armA=COMPLETE,blaADC-11=COMPLETE,blaADC-176=COMPLETE,blaOXA-23=COMPLETE,blaOXA-69=COMPLETE,mph(E)=COMPLETE,msr(E)=COMPLETE
TG22214	1	USA	sputum	clinical	2010	SAMN02055528	GCA_000453205.1	Contig	3.90282	38.9		ASFX01	85	85	195243	abaF=COMPLETE,adeC=COMPLETE,amvA=COMPLETE,ant(3'')-IIa=COMPLETE,aph(3')-Ia=COMPLETE,armA=COMPLETE,blaADC-11=COMPLETE,blaADC-176=COMPLETE,blaOXA-23=COMPLETE,blaOXA-69=COMPLETE,mph(E)=COMPLETE,msr(E)=COMPLETE,sul1=PARTIAL_END_OF_CONTIG
Canada BC-5	1	Canada	nosocomial spread of	clinical	2007	SAMN02436468	GCA_000286615.1	Contig	3.99802	39.1895	plasmid pCa	AFDN01	3	3	3211753	aac(3)-Ia=COMPLETE,aadA1=COMPLETE,abaF=COMPLETE,adeC=COMPLETE,amvA=COMPLETE,ant(3'')-IIa=COMPLETE,aph(3')-Ia=COMPLETE,blaADC-11=COMPLETE,blaADC=PARTIAL,blaOXA-23=COMPLETE,blaOXA-69=COMPLETE,blaOXA=COMPLETE,catA1=COMPLETE,sul1=COMPLETE,tet(A)=COMPLETE
1605	1	USA		environmental/other		SAMN02436567	GCA_000441955.1	Contig	3.94475	39.1		AUWL01	170	170	81601	aac(3)-Ia=COMPLETE,aadA1=COMPLETE,abaF=COMPLETE,adeC=COMPLETE,amvA=COMPLETE,ant(3'')-IIa=COMPLETE,aph(3')-Ia=COMPLETE,blaADC-11=COMPLETE,blaOXA-23=COMPLETE,blaOXA-69=COMPLETE,blaTEM-1=COMPLETE,catA1=COMPLETE,sul1=COMPLETE,tet(A)=COMPLETE
MRSN 57	1	USA	wound	clinical	2010-06	SAMN02437318	GCA_000586635.1	Contig	3.95346	39.1		JABT01	104	104	154990	aac(3)-Ia=COMPLETE,aadA1=PARTIAL_END_OF_CONTIG,abaF=COMPLETE,adeC=COMPLETE,amvA=COMPLETE,ant(3'')-IIa=COMPLETE,aph(3')-Ia=COMPLETE,aph(6)-Id=PARTIAL_END_OF_CONTIG,blaADC-191=COMPLETE,blaOXA-23=COMPLETE,blaOXA-69=COMPLETE,dfrA1=COMPLETE,sat2=COMPLETE,sul1=COMPLETE,sul2=COMPLETE,tet(B)=COMPLETE
MRSN 58	1	USA	wound	clinical	2010-06	SAMN02437332	GCA_000586655.1	Contig	3.9493	39.1		JABU01	104	104	145015	aac(3)-Ia=COMPLETE,aadA1=PARTIAL_END_OF_CONTIG,abaF=COMPLETE,adeC=COMPLETE,amvA=COMPLETE,ant(3'')-IIa=COMPLETE,aph(3')-Ia=COMPLETE,aph(6)-Id=PARTIAL_END_OF_CONTIG,blaADC-191=COMPLETE,blaOXA-23=COMPLETE,blaOXA-69=COMPLETE,dfrA1=COMPLETE,sat2=COMPLETE,sul1=COMPLETE,sul2=COMPLETE,tet(B)=COMPLETE
ABNIH11	1	USA	tracheal aspirate	clinical	2009	SAMN02470682	GCA_000354015.1	Contig	3.96818	39.1		APBA01	238	238	36816	aac(3)-Ia=COMPLETE,aadA1=COMPLETE,abaF=COMPLETE,adeC=COMPLETE,amvA=COMPLETE,ant(3'')-IIa=COMPLETE,aph(3')-Ia=COMPLETE,aph(6)-Id=PARTIAL,blaADC-191=COMPLETE,blaOXA-23=COMPLETE,blaOXA-69=COMPLETE,dfrA1=COMPLETE,sat2=COMPLETE,sul1=COMPLETE,sul2=COMPLETE,tet(B)=COMPLETE
ABNIH19	1	USA	tracheal aspirate	clinical	2009	SAMN02470684	GCA_000353775.1	Contig	3.98233	39.2		APBH01	160	160	66616	aac(3)-Ia=COMPLETE,aadA1=PARTIAL_END_OF_CONTIG,abaF=COMPLETE,adeC=COMPLETE,amvA=COMPLETE,ant(3'')-IIa=COMPLETE,aph(3')-Ia=COMPLETE,aph(3')-VIa=PARTIAL_END_OF_CONTIG,aph(6)-Id=PARTIAL,blaADC-191=COMPLETE,blaOXA-23=COMPLETE,blaOXA-69=COMPLETE,dfrA1=COMPLETE,sat2=COMPLETE,sul1=COMPLETE,sul2
ABNIH6	1	USA	bronchoalveolar lavag	clinical	2009	SAMN02470695	GCA_000353955.1	Contig	3.99105	39.1		APAX01	175	175	50876	aac(3)-Ia=COMPLETE,aadA1=COMPLETE,aadA1=PARTIAL_END_OF_CONTIG,abaF=COMPLETE,adeC=PARTIAL,amvA=COMPLETE,ant(3'')-IIa=COMPLETE,aph(3')-Ia=COMPLETE,aph(3')-VIa=COMPLETE,aph(3')-VIa=PARTIAL_END_OF_CONTIG,aph(6)-Id=PARTIAL,blaADC-191=COMPLETE,blaOXA-23=COMPLETE,blaOXA-69=COMPLETE,dfrA1=COMPLETE,sat2=COMPLETE,sul1
AB_908-13	1		urine	clinical		SAMN02471253	GCA_000302035.1	Contig	3.97316	38.9		AMHW01	175	175	75548	abaF=COMPLETE,adeC=COMPLETE,amvA=COMPLETE,ant(2'')-Ia=COMPLETE,ant(3'')-IIa=COMPLETE,aph(3')-VIa=COMPLETE,blaADC-11=COMPLETE,blaADC-176=COMPLETE,blaOXA-235=COMPLETE,blaOXA-23=COMPLETE,blaOXA-69=COMPLETE
AB_909-02-7	1		sputum	clinical		SAMN02471282	GCA_000301435.1	Contig	3.92964	38.9		AMHZ01	98	98	138985	abaF=COMPLETE,adeC=COMPLETE,amvA=COMPLETE,ant(3'')-IIa=COMPLETE,blaADC=PARTIAL_END_OF_CONTIG,blaOXA-23=COMPLETE,blaOXA-69=COMPLETE
AB0057	1	USA		environmental/other		SAMN02603051	GCA_000021245.2	Complete	4.06388	39.1897	chromosome:NC_011586.		2	2	4055148	aac(3)-Ia=COMPLETE,aadA1=COMPLETE,abaF=COMPLETE,adeC=COMPLETE,amvA=COMPLETE,ant(3'')-IIa=COMPLETE,aph(3')-Ia=COMPLETE,blaADC-11=COMPLETE,blaADC-176=COMPLETE,blaOXA-23=COMPLETE,blaOXA-69=COMPLETE,blaTEM-1=COMPLETE,catA1=COMPLETE,sul1=COMPLETE,tet(A)=COMPLETE
R1B	1	Saudi Arabia: Riyadh Armed Forces Hospital, Riy		clinical	3/1/11	SAMN02628532	GCA_000623035.1	Scaffold	4.25481	38.9		JICK01	23	39	449959	abaF=COMPLETE,adeC=COMPLETE,amvA=COMPLETE,ant(3'')-IIa=COMPLETE,aph(3')-VIa=COMPLETE,blaADC-11=COMPLETE,blaOXA-23=COMPLETE,blaOXA-69=COMPLETE,blaOXA=PARTIAL,mph(E)=COMPLETE,msr(E)=COMPLETE,sul2=COMPLETE,tet(39)=PARTIAL
AB5075	1	USA	bone culture	clinical	9/1/08	SAMN02713669	GCA_000770605.1	Contig	3.99987	38.8896	plasmid pAB	JHUI01	16	16	599779	aac(6')-Ib3=COMPLETE,aadA2=COMPLETE,abaF=COMPLETE,adeC=COMPLETE,amvA=COMPLETE,ant(2'')-Ia=COMPLETE,ant(3'')-IIa=COMPLETE,aph(3'')-Ib=COMPLETE,aph(3')-VIa=COMPLETE,aph(6)-Id=COMPLETE,blaADC-11=COMPLETE,blaGES-11=COMPLETE,blaOXA-23=COMPLETE,blaOXA-69=COMPLETE,cmlA1=COMPLETE,dfrA7=COMPLETE,sul1=COMPLETE
MRSN 3405	94	USA: Maryland	wound	clinical	3/29/11	SAMN02836944	GCA_000731975.1	Contig	4.02748	38.8		JNOU01	143	143	59081	abaF=COMPLETE,adeC=COMPLETE,amvA=COMPLETE,ant(2'')-Ia=COMPLETE,ant(3'')-IIa=COMPLETE,aph(3')-Ia=COMPLETE,aph(3')-VIa=COMPLETE,blaADC=COMPLETE,blaOXA-23=COMPLETE,blaOXA-69=COMPLETE,mph(E)=COMPLETE,msr(E)=COMPLETE,sul2=COMPLETE
MRSN 3405	94	USA	wound	clinical	2011-03	SAMN02906922	GCA_001028385.1	Contig	4.08272	38.9006	plasmid pMI	JPIA01	64	64	185299	abaF=COMPLETE,adeC=COMPLETE,amvA=COMPLETE,ant(2'')-Ia=COMPLETE,ant(3'')-IIa=COMPLETE,aph(3')-Ia=COMPLETE,aph(3')-VIa=COMPLETE,blaADC=COMPLETE,blaOXA-23=COMPLETE,blaOXA-69=COMPLETE,mph(E)=COMPLETE,msr(E)=COMPLETE,sul2=COMPLETE
MRSN 3527	81	USA	wound	clinical	2011-04	SAMN02906923	GCA_001028325.1	Contig	4.20619	38.9006	plasmid pMI	JPHZ01	46	46	218174	abaF=COMPLETE,adeC=COMPLETE,amvA=COMPLETE,ant(2'')-Ia=COMPLETE,ant(3'')-IIa=COMPLETE,aph(3')-VIa=COMPLETE,blaADC=COMPLETE,blaOXA-23=COMPLETE,blaOXA-69=COMPLETE,sul2=COMPLETE
MRSN 3942	94	USA	wound	clinical	2011-07	SAMN02906926	GCA_001028335.1	Contig	3.97572	39.0005	plasmid pMI	JPHY01	69	69	148854	abaF=COMPLETE,adeC=COMPLETE,amvA=COMPLETE,ant(2'')-Ia=COMPLETE,ant(3'')-IIa=COMPLETE,aph(3')-Ia=COMPLETE,aph(3')-VIa=COMPLETE,blaADC=PARTIAL_END_OF_CONTIG,blaOXA-23=COMPLETE,blaOXA-69=COMPLETE,mph(E)=COMPLETE,msr(E)=COMPLETE,sul2=COMPLETE
MRSN 4106	94	USA	wound	clinical	2011-07	SAMN02906927	GCA_001028305.1	Contig	3.95268	39.0005	plasmid pMI	JPHX01	62	62	173602	abaF=COMPLETE,adeC=COMPLETE,amvA=COMPLETE,ant(2'')-Ia=COMPLETE,ant(3'')-IIa=COMPLETE,aph(3')-Ia=COMPLETE,blaADC=COMPLETE,blaOXA-23=COMPLETE,blaOXA-69=COMPLETE,mph(E)=COMPLETE,msr(E)=COMPLETE,sul2=COMPLETE
MRSN 58	1	USA	wound	clinical	2010-06	SAMN02906928	GCA_001028315.1	Contig	3.97418	39.0888	plasmid pMI	JPHW01	40	40	247116	aac(3)-Ia=COMPLETE,aadA1=COMPLETE,abaF=COMPLETE,adeC=COMPLETE,amvA=COMPLETE,ant(3'')-IIa=COMPLETE,aph(3')-Ia=COMPLETE,aph(6)-Id=PARTIAL,blaADC-191=COMPLETE,blaOXA-23=COMPLETE,blaOXA-69=COMPLETE,dfrA1=COMPLETE,sat2=COMPLETE,sul1=COMPLETE,sul2=COMPLETE,tet(B)=COMPLETE
MRSN 7339	1	USA	wound	clinical	2004-09	SAMN02906929	GCA_001028265.1	Contig	3.95547	39.0894	plasmid pMI	JPHV01	34	34	246634	aac(3)-Ia=COMPLETE,aadA1=COMPLETE,abaF=COMPLETE,adeC=COMPLETE,amvA=COMPLETE,ant(3'')-IIa=COMPLETE,aph(3')-Ia=COMPLETE,blaADC=PARTIAL_END_OF_CONTIG,blaOXA-23=COMPLETE,blaOXA-69=COMPLETE,blaTEM-1=COMPLETE,catA1=COMPLETE,sul1=COMPLETE,tet(A)=COMPLETE
AB3340	20	USA: Walter Reed Army M	blood	clinical	10/22/06	SAMN03078661	GCA_001612005.1	Scaffold	4.01025	38.9		LRDU01	76	78	131957	abaF=COMPLETE,adeC=COMPLETE,amvA=COMPLETE,ant(3'')-IIa=COMPLETE,blaADC-74=COMPLETE,blaOXA-23=COMPLETE,blaOXA-69=COMPLETE,mph(E)=COMPLETE,msr(E)=COMPLETE,sul1=COMPLETE
AB3927	717	USA: Walter Reed Army M	Severe Trauma Site	clinical	5/18/07	SAMN03078666	GCA_001612115.1	Scaffold	4.11378	38.8		LRDZ01	45	47	227995	abaF=COMPLETE,adeC=COMPLETE,amvA=COMPLETE,ant(2'')-Ia=COMPLETE,ant(3'')-IIa=COMPLETE,aph(3')-Ia=COMPLETE,aph(3')-VIa=COMPLETE,aph(6)-Id=COMPLETE,blaADC=COMPLETE,blaOXA-69=COMPLETE,sul2=COMPLETE
AB4448	1	USA: Walter Reed Army M	warwound (surv)	clinical	12/25/07	SAMN03078672	GCA_001612255.1	Scaffold	3.99226	38.9		LREE01	43	44	354852	aac(6')-Ib3=COMPLETE,aadA2=COMPLETE,abaF=COMPLETE,adeC=COMPLETE,amvA=COMPLETE,ant(2'')-Ia=COMPLETE,ant(3'')-IIa=COMPLETE,aph(3'')-Ib=COMPLETE,aph(3')-VIa=COMPLETE,aph(6)-Id=COMPLETE,blaADC-11=COMPLETE,blaGES-11=COMPLETE,blaOXA-23=COMPLETE,blaOXA-69=COMPLETE,cmlA1=COMPLETE,dfrA7=COMPLETE,sul1=COMPLETE
AB4490	1	USA: Walter Reed Army M	warwound (surv)	clinical	1/12/08	SAMN03078674	GCA_001612275.1	Scaffold	3.9474	39		LREG01	98	104	84680	abaF=COMPLETE,adeC=COMPLETE,amvA=COMPLETE,ant(3'')-IIa=COMPLETE,blaADC-11=COMPLETE,blaOXA-23=COMPLETE,blaOXA-69=COMPLETE,sul1=COMPLETE
AB4991	1	USA: Walter Reed Army M	warwound (surv)	clinical	8/3/08	SAMN03078680	GCA_001612415.1	Scaffold	3.87711	39.1		LREM01	52	56	310788	aac(3)-Ia=COMPLETE,aadA1=COMPLETE,abaF=COMPLETE,adeC=COMPLETE,amvA=COMPLETE,ant(3'')-IIa=COMPLETE,aph(3')-Ia=COMPLETE,blaADC-176=COMPLETE,blaOXA-23=COMPLETE,blaOXA-69=COMPLETE,blaTEM=MISTRANSLATION,catA1=COMPLETE,sul1=COMPLETE,tet(A)=COMPLETE
AB5197	1	USA: Walter Reed Army M	Severe Trauma Site	clinical	10/15/08	SAMN03078682	GCA_001612455.1	Scaffold	3.95948	39		LREO01	58	63	158861	aac(3)-Ia=COMPLETE,abaF=COMPLETE,adeC=COMPLETE,amvA=COMPLETE,ant(3'')-IIa=COMPLETE,aph(3')-Ia=COMPLETE,aph(6)-Id=PARTIAL_END_OF_CONTIG,blaADC-191=COMPLETE,blaOXA-23=COMPLETE,blaOXA-69=COMPLETE,dfrA1=COMPLETE,sat2=COMPLETE,sul1=COMPLETE,sul2=COMPLETE,tet(B)=COMPLETE
AB5674	1	USA: Walter Reed Army M	blood	clinical	5/22/09	SAMN03078683	GCA_001612435.1	Scaffold	3.86925	39		LREP01	34	36	419504	abaF=COMPLETE,adeC=COMPLETE,amvA=COMPLETE,ant(3'')-IIa=COMPLETE,blaADC-11=COMPLETE,blaOXA-23=COMPLETE,blaOXA-69=COMPLETE,sul1=COMPLETE
6870155	1	Australia	Human sputum	clinical	2002	SAMN03105183	GCA_000794125.1	Scaffold	3.9814	39.3		JRWO01	19	115	96901	aac(3)-Ia=COMPLETE,aadA1=COMPLETE,abaF=COMPLETE,adeC=COMPLETE,amvA=COMPLETE,ant(3'')-IIa=COMPLETE,aph(3')-Ia=COMPLETE,blaADC-176=COMPLETE,blaADC=COMPLETE,blaOXA-69=COMPLETE,blaTEM-1=COMPLETE,catA1=COMPLETE,sul1=COMPLETE,tet(A)=COMPLETE
A1	1	United Kingdom: Nottingham		clinical	1982	SAMN03248539	GCA_000830055.1	Complete	3.91774	39.2891	chromosome:NZ_CP01078		2	2	3909008	aac(3)-Ia=COMPLETE,aadA1=COMPLETE,abaF=COMPLETE,adeC=COMPLETE,amvA=COMPLETE,ant(3'')-IIa=COMPLETE,blaADC-11=COMPLETE,blaOXA-69=COMPLETE,catA1=COMPLETE,sul1=COMPLETE,tet(A)=COMPLETE
A155	1	Argentina: Ciudad Autono	urine	clinical	1994-06	SAMN03276491	GCA_000836625.1	Contig	3.93345	39.1		JXSV01	53	53	197844	aac(3)-IIe=COMPLETE,aac(6')-Ib3=COMPLETE,abaF=COMPLETE,adeC=COMPLETE,amvA=COMPLETE,ant(3'')-IIa=COMPLETE,aph(3')-Ia=COMPLETE,blaADC=COMPLETE,blaOXA-69=COMPLETE,blaTEM-1=COMPLETE,catA1=COMPLETE,sul1=COMPLETE,tet(A)=COMPLETE
S36	1	Singapore		environmental/other	2010	SAMN03418031	GCA_000969385.1	Contig	4.02184	38.9		LAIO01	66	66	191442	aac(3)-Ia=COMPLETE,aadA1=COMPLETE,abaF=COMPLETE,adeC=COMPLETE,amvA=COMPLETE,ant(3'')-IIa=COMPLETE,aph(3')-VIa=COMPLETE,blaADC-176=COMPLETE,blaOXA-23=COMPLETE,blaOXA-69=COMPLETE,sul1=COMPLETE
AR_0045	1			clinical		SAMN04014886	GCA_001874945.1	Contig	4.09526	39.2		MPBW01	15	15	2075113	aac(3)-Ia=COMPLETE,aadA1=COMPLETE,abaF=COMPLETE,adeC=COMPLETE,amvA=COMPLETE,ant(3'')-IIa=COMPLETE,aph(3')-Ia=COMPLETE,blaADC-11=COMPLETE,blaADC-176=COMPLETE,blaOXA-23=COMPLETE,blaOXA-69=COMPLETE,blaTEM-1=COMPLETE,catA1=COMPLETE,sul1=COMPLETE,tet(A)=COMPLETE
AR_0083	1			clinical		SAMN04014924	GCA_002996805.1	Complete	4.15817	39.2897	chromosome:NZ_CP02752		2	2	4149444	aac(3)-Ia=COMPLETE,aadA1=COMPLETE,abaF=COMPLETE,adeC=COMPLETE,amvA=COMPLETE,ant(3'')-IIa=COMPLETE,aph(3'')-Ib=COMPLETE,aph(3')-Ia=COMPLETE,aph(6)-Id=PARTIAL,armA=COMPLETE,arr-2=COMPLETE,blaADC=COMPLETE,blaNDM-1=COMPLETE,blaOXA-23=COMPLETE,blaOXA-69=COMPLETE,blaPER-7=COMPLETE,ble=COMPLETE,
D36	81	Australia: Sydney	wound	clinical	2008	SAMN04029125	GCA_001399655.1	Complete	4.13116	39.0206	chromosome:NZ_CP01295		5	5	4063596	abaF=COMPLETE,adeC=COMPLETE,amvA=COMPLETE,ant(2'')-Ia=COMPLETE,ant(3'')-IIa=COMPLETE,aph(3')-Ia=COMPLETE,blaADC=COMPLETE,blaOXA-23=COMPLETE,blaOXA-69=COMPLETE,sul2=COMPLETE
MRSN4119	94	USA: Maryland, Bethesda	sacrum	clinical	2011-06	SAMN04254727	GCA_001443265.1	Contig	3.94602	38.9		LNBZ01	113	113	87985	abaF=COMPLETE,adeC=COMPLETE,amvA=COMPLETE,ant(2'')-Ia=COMPLETE,ant(3'')-IIa=COMPLETE,aph(3')-Ia=COMPLETE,aph(3')-VIa=COMPLETE,blaADC=COMPLETE,blaOXA-23=COMPLETE,blaOXA-69=COMPLETE,mph(E)=COMPLETE,msr(E)=COMPLETE,sul2=COMPLETE
MRSN3941	94	USA: Maryland, Bethesda	leg	clinical	2011-07	SAMN04254728	GCA_001444215.1	Contig	3.94996	38.9		LNCV01	112	112	79571	abaF=COMPLETE,adeC=COMPLETE,amvA=COMPLETE,ant(2'')-Ia=COMPLETE,ant(3'')-IIa=COMPLETE,aph(3')-Ia=COMPLETE,aph(3')-VIa=COMPLETE,blaADC=COMPLETE,blaOXA-23=COMPLETE,blaOXA-69=COMPLETE,mph(E)=COMPLETE,msr(E)=COMPLETE,sul2=COMPLETE
MRSN5540	94	USA: Maryland, Bethesda	blood	clinical	2011-01	SAMN04254729	GCA_001444225.1	Contig	4.02865	38.8		LNCW01	117	117	87980	abaF=COMPLETE,adeC=COMPLETE,amvA=COMPLETE,ant(2'')-Ia=COMPLETE,ant(3'')-IIa=COMPLETE,aph(3')-Ia=COMPLETE,aph(3')-VIa=COMPLETE,blaADC=COMPLETE,blaOXA-23=COMPLETE,blaOXA-69=COMPLETE,mph(E)=COMPLETE,msr(E)=COMPLETE,sul2=COMPLETE
MRSN6269	94	USA: Maryland, Bethesda	sputum	clinical	2012-01	SAMN04254730	GCA_001444255.1	Contig	4.02594	38.8		LNCX01	117	117	87286	abaF=COMPLETE,adeC=COMPLETE,amvA=COMPLETE,ant(2'')-Ia=COMPLETE,ant(3'')-IIa=COMPLETE,aph(3')-Ia=COMPLETE,aph(3')-VIa=COMPLETE,blaADC=COMPLETE,blaOXA-23=COMPLETE,blaOXA-69=COMPLETE,mph(E)=COMPLETE,msr(E)=COMPLETE,sul2=COMPLETE
MRSN6273	94	USA: Maryland, Bethesda	sputum	clinical	2012-01	SAMN04254731	GCA_001444265.1	Contig	3.95148	38.9		LNCY01	111	111	93592	abaF=COMPLETE,adeC=COMPLETE,amvA=COMPLETE,ant(2'')-Ia=COMPLETE,ant(3'')-IIa=COMPLETE,aph(3')-Ia=COMPLETE,aph(3')-VIa=COMPLETE,blaADC=COMPLETE,blaOXA-23=COMPLETE,blaOXA-69=COMPLETE,mph(E)=COMPLETE,msr(E)=COMPLETE,sul2=COMPLETE
AC001	20	Switzerland: Geneva	blood	clinical	2006	SAMN04272864	GCA_002416345.1	Contig	4.07587	38.9		LNUY01	70	70	132413	abaF=COMPLETE,adeC=COMPLETE,amvA=COMPLETE,ant(2'')-Ia=COMPLETE,ant(3'')-IIa=COMPLETE,blaADC-74=COMPLETE,blaOXA-23=COMPLETE,blaOXA-69=COMPLETE,mph(E)=COMPLETE,msr(E)=COMPLETE,sul1=COMPLETE
AC001-8-R1	20	Switzerland: Geneva	blood	clinical	2015	SAMN04272867	GCA_002416475.1	Contig	4.07808	38.9		LNVB01	75	75	132167	abaF=COMPLETE,adeC=COMPLETE,amvA=COMPLETE,ant(2'')-Ia=COMPLETE,ant(3'')-IIa=COMPLETE,blaADC-74=COMPLETE,blaOXA-23=COMPLETE,blaOXA-69=COMPLETE,mph(E)=COMPLETE,msr(E)=COMPLETE,sul1=COMPLETE
AC001-8-R1-S1	20	Switzerland: Geneva	blood	clinical	2015	SAMN04272868	GCA_002416375.1	Contig	4.08767	38.9		LNVC01	116	116	111337	abaF=COMPLETE,adeC=COMPLETE,amvA=COMPLETE,ant(2'')-Ia=COMPLETE,ant(3'')-IIa=COMPLETE,blaADC-74=COMPLETE,blaOXA-23=COMPLETE,blaOXA-69=COMPLETE,mph(E)=COMPLETE,msr(E)=COMPLETE,sul1=COMPLETE
MEX11594	1	Mexico:Mexico City	Pleural fluid	clinical	6/17/11	SAMN04407353	GCA_001549575.1	Scaffold	3.7455	38.9873	plasmid unn	LQXZ01	12	12	684685	abaF=COMPLETE,adeC=COMPLETE,amvA=COMPLETE,ant(3'')-IIa=COMPLETE,blaADC-11=COMPLETE,blaOXA-69=COMPLETE
CRO1	1	Croatia	tracheal aspirate	clinical	2003	SAMN05229953	GCA_001907145.1	Contig	4.01824	38.9		LZYQ01	89	89	161990	aac(3)-Ia=COMPLETE,aadA1=COMPLETE,abaF=COMPLETE,adeC=COMPLETE,amvA=PARTIAL_END_OF_CONTIG,ant(3'')-IIa=COMPLETE,aph(3')-Ia=COMPLETE,aph(3')-VIa=PARTIAL_END_OF_CONTIG,blaADC=COMPLETE,blaOXA-107=COMPLETE,sul1=COMPLETE
CRO3	1	Croatia	tracheal aspirate	clinical	2013	SAMN05229955	GCA_001907155.1	Contig	3.96444	38.8		LZYS01	89	89	113309	aac(3)-Ia=COMPLETE,aadA1=COMPLETE,abaF=COMPLETE,adeC=COMPLETE,amvA=COMPLETE,ant(3'')-IIa=COMPLETE,aph(3')-VIa=COMPLETE,blaADC-74=COMPLETE,blaOXA-69=COMPLETE,blaOXA-72=COMPLETE,sul1=COMPLETE
71	81	Spain:Madrid	blood	clinical	1/5/10	SAMN05590239	GCA_002119265.1	Scaffold	3.95183	39		MEDG01	57	57	139653	aac(6')-Ib3=COMPLETE,abaF=COMPLETE,adeC=COMPLETE,amvA=COMPLETE,ant(2'')-Ia=COMPLETE,ant(3'')-IIa=COMPLETE,aph(3')-Ia=COMPLETE,blaADC-11=COMPLETE,blaOXA-69=COMPLETE,sul1=COMPLETE
440	81	Spain:Madrid	blood	clinical	1/21/10	SAMN05590405	GCA_002119355.1	Scaffold	3.95771	39		MEDH01	54	54	156521	aac(6')-Ib3=COMPLETE,abaF=COMPLETE,adeC=COMPLETE,amvA=COMPLETE,ant(2'')-Ia=COMPLETE,ant(3'')-IIa=COMPLETE,blaADC-11=COMPLETE,blaOXA-58=COMPLETE,blaOXA-69=COMPLETE,sul1=COMPLETE
USA15	1	South Korea: Seoul	sputum	clinical	2013-10	SAMN06650240	GCA_002082825.1	Complete	4.02848	39.078	chromosome:NZ_CP0205		2	2	3930178	abaF=PARTIAL,adeC=COMPLETE,amvA=COMPLETE,ant(3'')-IIa=COMPLETE,aph(3')-VIa=COMPLETE,blaADC-11=COMPLETE,blaADC-176=COMPLETE,blaOXA-23=COMPLETE,blaOXA-69=COMPLETE,sul1=COMPLETE
PR332	81			clinical		SAMN06837753	GCA_002137655.1	Contig	4.04705	38.9		NGDV01	64	64	196528	abaF=COMPLETE,adeC=COMPLETE,amvA=COMPLETE,ant(2'')-Ia=COMPLETE,ant(3'')-IIa=COMPLETE,blaADC=COMPLETE,blaOXA-69=COMPLETE,sul2=COMPLETE
ARLG1314	1			clinical		SAMN06892213	GCA_002143965.1	Contig	4.03699	39		NGHN01	73	73	347166	aac(3)-Ia=COMPLETE,aadA1=COMPLETE,abaF=COMPLETE,adeC=COMPLETE,amvA=COMPLETE,ant(3'')-IIa=COMPLETE,aph(3')-Ia=COMPLETE,blaADC=PARTIAL_END_OF_CONTIG,blaOXA-23=COMPLETE,blaOXA-69=COMPLETE,blaTEM-1=COMPLETE,catA1=COMPLETE,sul1=COMPLETE,tet(A)=COMPLETE
A85	1	Australia: Sydney	sputum	clinical	2003	SAMN07125723	GCA_002210065.1	Complete	4.14461	39.1765	chromosome:NZ_CP02178		6	6	4039997	aac(3)-Ia=COMPLETE,aadA1=COMPLETE,abaF=COMPLETE,adeC=COMPLETE,amvA=COMPLETE,ant(3'')-IIa=COMPLETE,aph(3')-Ia=COMPLETE,blaADC-11=COMPLETE,blaADC-176=COMPLETE,blaOXA-23=COMPLETE,blaOXA-69=COMPLETE,blaTEM-1=COMPLETE,catA1=COMPLETE,sul1=COMPLETE,tet(A)=COMPLETE
13	1	Brazil: Uberlandia, MG	tracheal aspirate	clinical	2/2/12	SAMN07257378	GCA_002245595.1	Scaffold	3.93866	38.9		NKKO01	104	104	100313	aac(6')-Ib=COMPLETE,abaF=COMPLETE,adeC=COMPLETE,amvA=COMPLETE,ant(3'')-IIa=COMPLETE,blaADC=COMPLETE,blaOXA-23=COMPLETE,blaOXA-69=COMPLETE
15	1	Brazil	bedside table	environmental/other	10/18/11	SAMN07286190	GCA_002245665.1	Scaffold	3.87756	38.9		NKKP01	248	248	35939	aac(6')-Ib=COMPLETE,abaF=COMPLETE,adeC=COMPLETE,amvA=COMPLETE,ant(3'')-IIa=COMPLETE,aph(3'')-Ib=COMPLETE,aph(6)-Id=COMPLETE,blaADC=COMPLETE,blaOXA-23=COMPLETE,blaOXA-69=COMPLETE,floR=COMPLETE,sul2=COMPLETE
IHS3526	1	Honduras: Cortes, San Pec	Endobronchial tube	clinical	2/11/16	SAMN07303764	GCA_002927895.1	Scaffold	3.78279	39.4		NQXR01	51	53	237336	aac(3)-Ia=COMPLETE,aadA1=COMPLETE,adeC=COMPLETE,amvA=COMPLETE,ant(3'')-IIa=COMPLETE,aph(3')-VIa=COMPLETE,blaADC=COMPLETE,blaCTX-M-15=COMPLETE,blaOXA-69=COMPLETE,catA1=COMPLETE,sul1=COMPLETE,tet(A)=COMPLETE
Abau446	1	Togo: Lome	pus	clinical	8/8/16	SAMN07514514	GCA_014334865.1	Scaffold	3.94701	39.2		NRID01	169	214	51406	aac(3)-Ia=COMPLETE,aadA1=PARTIAL_END_OF_CONTIG,abaF=COMPLETE,adeC=PARTIAL_END_OF_CONTIG,amvA=COMPLETE,ant(3'')-IIa=COMPLETE,aph(3'')-Ib=COMPLETE,aph(3')-Ia=COMPLETE,aph(3')-VIa=COMPLETE,aph(6)-Id=PARTIAL_END_OF_CONTIG,blaADC-191=COMPLETE,blaNDM-1=COMPLETE,blaOXA-23=COMPLETE,blaOXA-69=COMPLETE,ble=COMPLETE
9102	1	Mexico: Baja California	Bronchial fluid	clinical	6/4/10	SAMN07520232	GCA_003522845.1	Complete	4.06683	38.9829	chromosome:NZ_CP02302		2	2	3971623	abaF=COMPLETE,adeC=COMPLETE,amvA=COMPLETE,ant(3'')-IIa=COMPLETE,aph(3'')-Ib=COMPLETE,aph(3')-VIa=COMPLETE,aph(6)-Id=COMPLETE,blaADC=COMPLETE,blaOXA-235=COMPLETE,blaOXA-69=COMPLETE,sul2=COMPLETE
A388	1	Greece	not recorded	clinical	2002	SAMN07736509	GCA_002741415.1	Complete	4.03205	39.2869	chromosome:NZ_CP02441		2	2	3999012	aac(3)-Ia=COMPLETE,aac(6')-Ib3=COMPLETE,aadA1=COMPLETE,abaF=COMPLETE,adeC=COMPLETE,amvA=COMPLETE,ant(3'')-IIa=COMPLETE,aph(3')-Ia=COMPLETE,aph(3')-VIa=COMPLETE,blaADC-81=COMPLETE,blaOXA-58=COMPLETE,blaOXA-92=COMPLETE,blaTEM-1=COMPLETE,catA1=COMPLETE,sul1=COMPLETE,tet(A)=COMPLETE
AB307-0294	1	USA	blood	clinical	1994	SAMN07961492	GCA_002803025.2	Complete	3.75949	39	chromosome:NZ_CP00117		1	1	3759495	abaF=COMPLETE,adeC=COMPLETE,amvA=COMPLETE,ant(3'')-IIa=COMPLETE,blaADC-11=COMPLETE,blaOXA-69=COMPLETE
ZQ3	717	Iraq: Baghdad	blood	clinical	2016	SAMN08093362	GCA_002838025.2	Scaffold	4.10709	38.8832	plasmid p1Z	PHJZ02	73	78	128541	abaF=COMPLETE,adeC=COMPLETE,amvA=COMPLETE,ant(3'')-IIa=COMPLETE,blaADC=COMPLETE,blaOXA-23=COMPLETE,blaOXA-69=COMPLETE,sul2=COMPLETE
ZQ5	1	Iraq: Baghdad	wound swab	clinical	2016	SAMN08093364	GCA_002837955.2	Scaffold	4.04437	39.1845	plasmid p2Z	PHJX02	72	77	180690	aac(3)-Ia=COMPLETE,aadA1=COMPLETE,aadA1=PARTIAL_END_OF_CONTIG,abaF=COMPLETE,adeC=COMPLETE,amvA=COMPLETE,ant(3'')-IIa=COMPLETE,aph(3')-Ia=COMPLETE,aph(3')-VIa=COMPLETE,aph(6)-Id=PARTIAL,armA=COMPLETE,arr-2=COMPLETE,blaADC-191=COMPLETE,blaOXA-23=COMPLETE,blaOXA-69=COMPLETE,blaPER-7=COMPLETE,cmlA5=COMPLETE,(
ZQ6	1	Iraq: Baghdad	wound swab	clinical	2016	SAMN08093365	GCA_002838005.2	Scaffold	4.10559	39.0664	plasmid p1Z	PHJW02	90	99	159993	aac(3)-Ia=COMPLETE,aadA1=COMPLETE,abaF=COMPLETE,adeC=COMPLETE,amvA=COMPLETE,ant(3'')-IIa=COMPLETE,aph(3')-Ia=COMPLETE,aph(3')-VIa=COMPLETE,aph(6)-Id=PARTIAL,armA=COMPLETE,arr-2=COMPLETE,blaADC-191=COMPLETE,blaOXA-23=COMPLETE,blaOXA-69=COMPLETE,blaPER-7=COMPLETE,cmlA5=COMPLETE,dfrA1=COMPLETE,mph(E)=COMPLE
WCHAB005078	20	China: Sichuan, Chengdu		clinical		SAMN08364584	GCA_002999195.2	Complete	4.14005	39.2245	chromosome:NZ_CP02724		4	4	3990012	aac(3)-IId=COMPLETE,aadA2=COMPLETE,abaF=COMPLETE,adeC=COMPLETE,amvA=COMPLETE,ant(3'')-IIa=COMPLETE,armA=COMPLETE,blaADC-79=COMPLETE,blaOXA-58=COMPLETE,blaOXA-69=COMPLETE,catA1=COMPLETE,dfrA12=COMPLETE,floR=COMPLETE,mph(E)=COMPLETE,msr(E)=COMPLETE,sul1=COMPLETE,tet(A)=COMPLETE
A1-360	1	Brazil	cerebrospinal fluid	clinical	11/26/10	SAMN08383932	GCA_003325665.1	Contig	3.90277	39.1		QORH01	144	144	95482	aac(6')-Ib=COMPLETE,aadA1=COMPLETE,abaF=COMPLETE,adeC=COMPLETE,amvA=COMPLETE,ant(3'')-IIa=COMPLETE,blaADC-182=COMPLETE,blaOXA-23=COMPLETE,blaOXA-69=COMPLETE,sul1=COMPLETE
E1-143	1	Brazil	cerebrospinal fluid	clinical	2/18/14	SAMN08383996	GCA_003325575.1	Contig	3.95033	39		QORF01	159	159	80867	aac(3)-Ia=COMPLETE,aac(6')-Ib=COMPLETE,aadA1=COMPLETE,abaF=COMPLETE,adeC=COMPLETE,amvA=COMPLETE,ant(2'')-Ia=COMPLETE,ant(3'')-IIa=COMPLETE,aph(3')-Ia=COMPLETE,aph(3')-VIa=COMPLETE,blaADC-182=COMPLETE,blaOXA-23=COMPLETE,blaOXA-69=COMPLETE,floR=COMPLETE,sul1=COMPLETE,sul2=COMPLETE

SGH0410	1	Singapore: Singapore Gen	Endotracheal aspirati	clinical	2004	SAMN08637734	GCA_003020365.1	Contig	4.05472	39		PYEB01	73	73	463297	aac(3)-Ia=COMPLETE,aadA1=COMPLETE,abaF=COMPLETE,adeC=COMPLETE,amvA=COMPLETE,ant(3'')-IIa=COMPLETE,aph(3')-Ia=COMPLETE,blaADC-11=COMPLETE,blaADC=PARTIAL_END_OF_CONTIG,blaOXA-23=COMPLETE,blaOXA-69=COMPLETE,blaTEM-1=COMPLETE,catA1=COMPLETE,sul1=COMPLETE,tet(A)=COMPLETE
SGH0411	1	Singapore: Singapore Gen	Endotracheal aspirati	clinical	2004	SAMN08637735	GCA_003020415.1	Contig	3.91812	39		PYEA01	82	82	304305	aac(3)-Ia=COMPLETE,aadA1=COMPLETE,abaF=COMPLETE,adeC=COMPLETE,amvA=COMPLETE,ant(3'')-IIa=COMPLETE,aph(3')-Ia=COMPLETE,blaADC-11=COMPLETE,blaADC=PARTIAL_END_OF_CONTIG,blaOXA-23=COMPLETE,blaOXA-69=COMPLETE,sul1=COMPLETE
SGH0606	1	Singapore: Singapore Gen	Endotracheal aspirati	clinical	2006	SAMN08637736	GCA_003020215.1	Contig	4.10036	38.9		PYDZ01	106	106	251457	aac(3)-Ia=COMPLETE,aadA1=COMPLETE,abaF=COMPLETE,adeC=COMPLETE,amvA=COMPLETE,ant(3'')-IIa=COMPLETE,aph(3')-VIa=COMPLETE,blaADC=PARTIAL_END_OF_CONTIG,blaOXA-23=COMPLETE,blaOXA-69=COMPLETE,sul1=COMPLETE
SGH0807	1	Singapore: Singapore Gen	blood	clinical	2008	SAMN08637738	GCA_003020405.1	Contig	4.13513	39		PYDX01	139	139	279343	aac(3)-Ia=COMPLETE,aadA1=COMPLETE,abaF=PARTIAL,adeC=COMPLETE,amvA=COMPLETE,ant(3'')-IIa=COMPLETE,aph(3')-VIa=COMPLETE,blaADC-176=COMPLETE,blaADC=PARTIAL_END_OF_CONTIG,blaOXA-23=COMPLETE,blaOXA-69=COMPLETE,blaTEM-1=COMPLETE,catA1=COMPLETE,sul1=COMPLETE,tet(A)=COMPLETE
SGH0915	1	Singapore: Singapore Gen	sputum	clinical	2009	SAMN08637739	GCA_003020205.1	Contig	3.91771	38.9		PYDW01	81	81	239307	abaF=COMPLETE,adeC=COMPLETE,amvA=COMPLETE,ant(3'')-IIa=COMPLETE,blaADC-11=COMPLETE,blaADC=PARTIAL_END_OF_CONTIG,blaOXA-23=COMPLETE,blaOXA-69=COMPLETE,sul1=COMPLETE
SGH1101	1	Singapore: Singapore Gen	sputum	clinical	2011	SAMN08637740	GCA_003020225.1	Contig	4.00421	38.9		PYDV01	100	100	341578	aac(3)-Ia=COMPLETE,aadA1=COMPLETE,abaF=COMPLETE,adeC=COMPLETE,amvA=COMPLETE,ant(3'')-IIa=COMPLETE,aph(3')-Ia=COMPLETE,aph(3')-VIa=COMPLETE,blaADC-11=COMPLETE,blaADC=PARTIAL_END_OF_CONTIG,blaOXA-23=COMPLETE,blaOXA-69=COMPLETE,sul1=COMPLETE
5457	623	India		clinical		SAMN09244198	GCA_009428985.1	Chromosome	4.25055	39.1	chromosome:NZ_CP04554	1		1	4250446	abaF=COMPLETE,adeC=COMPLETE,amvA=COMPLETE,ant(3'')-IIa=COMPLETE,aph(3'')-Ib=COMPLETE,aph(3')-VIa=COMPLETE,aph(6)-Id=COMPLETE,blaADC=COMPLETE,blaOXA-23=COMPLETE,blaOXA-371=COMPLETE,floR=COMPLETE,mph(E)=COMPLETE,msr(E)=PARTIAL,sul2=COMPLETE
DA33382	1	Germany	Tracheal secretion	clinical		SAMN09460321	GCA_003296225.1	Complete	4.03682	38.9899	chromosome:NZ_CP03010	4		4	3947407	aac(3)-Ia=COMPLETE,aadA1=COMPLETE,abaF=COMPLETE,adeC=COMPLETE,amvA=COMPLETE,ant(3'')-IIa=COMPLETE,aph(3')-Ia=COMPLETE,aph(3')-VIa=COMPLETE,blaADC-186=COMPLETE,blaOXA-164=COMPLETE,blaOXA-69=COMPLETE,sul1=COMPLETE
VB29123	1	India: Vellore	blood	clinical	2017	SAMN10170275	GCA_003627465.1	Contig	4.04174	38.9		RBVT01	160	160	74961	abaF=COMPLETE,adeC=COMPLETE,amvA=COMPLETE,ant(3'')-IIa=COMPLETE,aph(3'')-Ib=COMPLETE,aph(3')-VIa=COMPLETE,aph(6)-Id=PARTIAL,blaADC=COMPLETE,blaNDM-1=COMPLETE,blaOXA-23=COMPLETE,blaOXA-371=COMPLETE,ble=COMPLETE,mph(E)=COMPLETE,msr(E)=COMPLETE,sul2=PARTIAL
AB_235	1	Pakistan	bedside light switch i	environmental/other	2016-02	SAMN10249015	GCA_003940075.1	Scaffold	4.03289	39		RHYP01	94	104	116757	aac(3)-Ia=COMPLETE,aadA1=COMPLETE,abaF=COMPLETE,adeC=COMPLETE,amvA=COMPLETE,ant(3'')-IIa=COMPLETE,aph(3')-Ia=COMPLETE,aph(3')-VIa=COMPLETE,blaOXA-23=COMPLETE,blaOXA-69=COMPLETE,catA1=COMPLETE,mph(E)=COMPLETE,msr(E)=COMPLETE,sul1=COMPLETE,tet(A)=COMPLETE
TG22592	1	USA: Arizona	BAL	clinical	10/15/11	SAMN10261541	GCA_003949635.1	Contig	3.95387	38.9		RFDX01	105	105	94732	abaF=COMPLETE,adeC=COMPLETE,amvA=COMPLETE,ant(3'')-IIa=COMPLETE,aph(3')-Ia=COMPLETE,armA=COMPLETE,blaADC=PARTIAL_END_OF_CONTIG,blaOXA-23=COMPLETE,blaOXA-69=COMPLETE,mph(E)=COMPLETE,msr(E)=COMPLETE,sul1=COMPLETE
TG29424	1	USA: Arizona	trach asp	clinical	3/6/12	SAMN10261559	GCA_003948995.1	Contig	3.83943	38.9		RFDF01	104	104	84552	abaF=COMPLETE,adeC=PARTIAL,amvA=COMPLETE,ant(3'')-IIa=COMPLETE,blaADC=PARTIAL_END_OF_CONTIG,blaOXA-23=COMPLETE,blaOXA-69=COMPLETE,mph(E)=COMPLETE,msr(E)=COMPLETE
TG22636	1	USA: Arizona	wound	clinical	10/15/11	SAMN10261577	GCA_003948695.1	Contig	3.95519	38.9		RFCN01	70	70	196920	abaF=COMPLETE,adeC=COMPLETE,amvA=COMPLETE,ant(3'')-IIa=COMPLETE,aph(3')-Ia=COMPLETE,armA=COMPLETE,blaADC=PARTIAL_END_OF_CONTIG,blaOXA-23=COMPLETE,blaOXA-69=COMPLETE,mph(E)=COMPLETE,msr(E)=COMPLETE
TG15482	1		sputum	clinical	11/8/10	SAMN10261598	GCA_003948175.1	Contig	3.94137	38.9		RFBS01	91	91	112592	abaF=COMPLETE,adeC=COMPLETE,amvA=COMPLETE,ant(3'')-IIa=COMPLETE,blaADC=PARTIAL_END_OF_CONTIG,blaOXA-23=COMPLETE,blaOXA-69=COMPLETE
VB24319	1	India: Vellore	blood	clinical	2017	SAMN10338982	GCA_003711885.1	Contig	4.05471	38.9		RHLV01	206	206	74409	abaF=COMPLETE,adeC=COMPLETE,amvA=COMPLETE,ant(3'')-IIa=COMPLETE,aph(3'')-Ib=COMPLETE,aph(3')-VI=PARTIAL_END_OF_CONTIG,aph(6)-Id=COMPLETE,blaADC=COMPLETE,blaNDM-1=COMPLETE,blaOXA-371=COMPLETE,ble=COMPLETE,mph(E)=COMPLETE,msr(E)=COMPLETE,sul2=PARTIAL
521_17	1	Brazil: Sao Paulo, Sao Pau	blood	clinical	2017	SAMN10797507	GCA_004347305.1	Contig	3.83441	38.9		SDHE01	57	57	127470	aac(6')-Ib=COMPLETE,adeC=COMPLETE,amvA=COMPLETE,ant(3'')-IIa=COMPLETE,aph(3')-VIa=COMPLETE,blaADC-235=COMPLETE,blaOXA-23=COMPLETE,blaOXA-69=COMPLETE,blaOXA=PARTIAL_END_OF_CONTIG,mph(E)=COMPLETE,msr(E)=COMPLETE
ABS201	328	Iran	Wound sample	clinical	2013	SAMN11626014	GCA_009268055.1	Contig	4.1981	38.8		VJZY01	125	125	89621	abaF=COMPLETE,amvA=COMPLETE,ant(3'')-IIa=COMPLETE,aph(3')-VIa=COMPLETE,blaADC=COMPLETE,blaOXA-23=COMPLETE,blaOXA-69=COMPLETE,blaOXA-72=COMPLETE,sul2=COMPLETE,tet(39)=COMPLETE
11W359501	1	United Kingdom: Nottingham		clinical	2015	SAMN12015779	GCA_006385075.1	Complete	4.13019	39.1	chromosome:NZ_CP04103	1		1	4130187	aac(3)-Ia=COMPLETE,aadA1=COMPLETE,abaF=COMPLETE,adeC=COMPLETE,amvA=COMPLETE,ant(3'')-IIa=COMPLETE,blaADC-184=COMPLETE,blaOXA-69=COMPLETE,sul1=COMPLETE,tet(A)=COMPLETE
MRSN15049	1	Afghanistan	respiratory	clinical	2013	SAMN12087641	GCA_006494155.1	Contig	3.89203	38.9		VHGW01	161	161	71950	abaF=COMPLETE,adeC=COMPLETE,amvA=COMPLETE,ant(3'')-IIa=COMPLETE,aph(3'')-Ib=COMPLETE,aph(3')-VIa=COMPLETE,aph(6)-Id=COMPLETE,blaADC=COMPLETE,blaOXA-23=COMPLETE,blaOXA-371=COMPLETE,sul2=COMPLETE
MRSN14193	1	Honduras	wound	clinical	2012	SAMN12087645	GCA_006494215.1	Contig	3.78383	39.1		VHGZ01	114	114	113518	aac(3)-Ia=COMPLETE,aadA1=COMPLETE,adeC=COMPLETE,amvA=COMPLETE,ant(3'')-IIa=COMPLETE,aph(3')-Ia=COMPLETE,aph(3')-VIa=COMPLETE,blaADC=PARTIAL_END_OF_CONTIG,blaCTX-M-15=COMPLETE,blaOXA-24=COMPLETE,blaOXA-69=COMPLETE,blaTEM-1=COMPLETE,catA1=COMPLETE,sul1=COMPLETE,tet(A)=COMPLETE
MRSN31196	20	Germany	groin	clinical	2015	SAMN12087664	GCA_006491855.1	Contig	4.05548	39		VHFU01	143	143	108897	aac(3)-Ia=COMPLETE,aadA1=COMPLETE,abaF=COMPLETE,amvA=COMPLETE,ant(3'')-IIa=COMPLETE,aph(3')-Ia=COMPLETE,aph(3')-VIa=COMPLETE,blaADC=COMPLETE,blaOXA-23=COMPLETE,blaOXA-69=COMPLETE,blaTEM-1=COMPLETE,catA1=COMPLETE,sul1=COMPLETE,tet(A)=COMPLETE
MRSN32108	1	Germany	wound	clinical	2006	SAMN12087674	GCA_006492525.1	Contig	4.04112	38.8		VHFK01	106	106	170579	abaF=COMPLETE,adeC=COMPLETE,amvA=COMPLETE,ant(2'')-Ia=COMPLETE,ant(3'')-IIa=COMPLETE,aph(3'')-Ib=COMPLETE,aph(3')-VIa=COMPLETE,aph(6)-Id=COMPLETE,blaADC=COMPLETE,blaOXA-69=COMPLETE,sul2=COMPLETE,tet(39)=COMPLETE
MRSN32865	94	Germany	wound	clinical	2007	SAMN12087678	GCA_006492735.1	Contig	3.92295	38.9		VHFF01	154	154	59653	abaF=COMPLETE,adeC=COMPLETE,amvA=COMPLETE,ant(2'')-Ia=COMPLETE,ant(3'')-IIa=COMPLETE,aph(3')-VIa=COMPLETE,blaADC=COMPLETE,blaOXA-69=COMPLETE,mph(E)=COMPLETE,msr(E)=COMPLETE,sul2=COMPLETE
MRSN489678	19	Ukraine	wound	clinical	2014	SAMN12087687	GCA_006492055.1	Contig	3.80111	38.9		VHEN01	125	125	77989	adeC=COMPLETE,amvA=COMPLETE,ant(2'')-Ia=COMPLETE,ant(3'')-IIa=COMPLETE,blaADC=COMPLETE,blaOXA-69=COMPLETE,sul2=COMPLETE
MRSN959	1	USA	wound	clinical	2008	SAMN12087695	GCA_006491985.1	Contig	3.98219	38.9		VHDS01	111	111	108645	aac(6')-Ib3=COMPLETE,aadA2=COMPLETE,abaF=COMPLETE,adeC=COMPLETE,amvA=COMPLETE,ant(2'')-Ia=COMPLETE,ant(3'')-IIa=COMPLETE,aph(3'')-Ib=COMPLETE,aph(3')-VIa=COMPLETE,aph(6)-Id=COMPLETE,blaADC-11=COMPLETE,blaGES-11=COMPLETE,blaOXA-23=COMPLETE,blaOXA-69=COMPLETE,cmlA1=COMPLETE,dfrA7=COMPLETE,sul1=COMPLETE
MRSN960	1	USA	wound	clinical	2008	SAMN12087696	GCA_006491965.1	Contig	3.94856	39		VHDR01	125	125	78227	aac(3)-Ia=COMPLETE,aadA1=PARTIAL_END_OF_CONTIG,abaF=COMPLETE,adeC=COMPLETE,amvA=COMPLETE,ant(3'')-IIa=COMPLETE,aph(3')-Ia=COMPLETE,aph(3')-VIa=COMPLETE,aph(6)-Id=PARTIAL_END_OF_CONTIG,blaADC-191=COMPLETE,blaOXA-23=COMPLETE,blaOXA-69=COMPLETE,dfrA1=COMPLETE,sat2=COMPLETE,sul1=COMPLETE,sul2=COMPLETE,tet(B)=C
MRSN1311	1	USA	blood	clinical	2007	SAMN12087702	GCA_006494165.1	Contig	3.95207	39		VHHA01	129	129	114894	aac(3)-Ia=COMPLETE,aadA1=COMPLETE,abaF=COMPLETE,adeC=COMPLETE,amvA=COMPLETE,ant(3'')-IIa=COMPLETE,aph(3')-Ia=COMPLETE,blaOXA-23=COMPLETE,blaOXA-69=COMPLETE,blaTEM-1=COMPLETE,catA1=COMPLETE,sul1=COMPLETE,tet(A)=COMPLETE
MRSN6541	1	USA	wound	clinical	2012	SAMN12087708	GCA_006492195.1	Contig	4.10286	39		VHEK01	156	156	107352	aadA2=COMPLETE,abaF=COMPLETE,amvA=COMPLETE,ant(2'')-Ia=COMPLETE,ant(3'')-IIa=COMPLETE,blaADC-11=COMPLETE,blaCARB-2=COMPLETE,blaOXA-23=COMPLETE,blaOXA-69=COMPLETE,catB11=COMPLETE,catB3=COMPLETE,cmlA1=COMPLETE,dfrA19=COMPLETE,mph(E)=COMPLETE,msr(E)=COMPLETE,sul1=COMPLETE
MRSN7067	81	USA	blood	clinical	2003	SAMN12087709	GCA_006492575.1	Contig	4.02621	38.9		VHEJ01	112	112	111760	abaF=COMPLETE,adeC=COMPLETE,amvA=COMPLETE,ant(2'')-Ia=COMPLETE,ant(3'')-IIa=COMPLETE,aph(3')-Ia=COMPLETE,blaADC=COMPLETE,blaOXA-69=COMPLETE,sul2=COMPLETE
MRSN7213	1	USA	wound	clinical	2003	SAMN12087714	GCA_006492065.1	Contig	3.7754	38.9		VHEE01	73	73	108743	abaF=COMPLETE,adeC=COMPLETE,amvA=COMPLETE,ant(2'')-Ia=COMPLETE,ant(3'')-IIa=COMPLETE,blaADC-25=COMPLETE,blaOXA-69=COMPLETE
2846	19	Georgia: Tbilisi		clinical	2018	SAMN12509150	GCA_009445405.1	Contig	3.90112	39.2		WIVM01	90	90	110424	abaF=COMPLETE,adeC=COMPLETE,amvA=COMPLETE,ant(3'')-IIa=COMPLETE,blaADC-185=COMPLETE,blaOXA-23=COMPLETE,blaOXA-69=COMPLETE,catA1=COMPLETE,sul2=COMPLETE,tet(B)=COMPLETE
ABS029	328	Iran	wound	clinical	2012	SAMN12726215	GCA_009497595.1	Contig	4.10299	38.9		WIOH01	106	106	102004	abaF=COMPLETE,amvA=COMPLETE,ant(3'')-IIa=COMPLETE,aph(3')-VIa=COMPLETE,blaADC=COMPLETE,blaOXA-23=COMPLETE,blaOXA-69=COMPLETE,blaOXA-72=COMPLETE,sul2=COMPLETE,tet(39)=COMPLETE
ABS042	328	Iran	wound	clinical	2012	SAMN12726216	GCA_009497615.1	Contig	4.17208	38.8		WIOG01	106	106	123947	abaF=COMPLETE,amvA=COMPLETE,ant(2'')-Ia=COMPLETE,ant(3'')-IIa=COMPLETE,aph(3')-VIa=COMPLETE,blaADC=COMPLETE,blaOXA-23=COMPLETE,blaOXA-69=COMPLETE,blaOXA-72=COMPLETE,sul2=COMPLETE
ABS062	328	Iran	wound	clinical	2012	SAMN12726217	GCA_009497575.1	Contig	4.10418	38.9		WIOF01	116	116	101391	abaF=COMPLETE,amvA=COMPLETE,ant(3'')-IIa=COMPLETE,aph(3')-VIa=COMPLETE,blaADC=COMPLETE,blaOXA-23=COMPLETE,blaOXA-69=COMPLETE,blaOXA-72=COMPLETE,sul2=COMPLETE,tet(39)=COMPLETE
ABS063	328	Iran	wound	clinical	2012	SAMN12726218	GCA_009497495.1	Contig	4.10217	38.9		WIOE01	109	109	102004	abaF=COMPLETE,amvA=COMPLETE,ant(3'')-IIa=COMPLETE,aph(3')-VIa=COMPLETE,blaADC=COMPLETE,blaOXA-23=COMPLETE,blaOXA-69=COMPLETE,blaOXA-72=COMPLETE,sul2=COMPLETE,tet(39)=COMPLETE
ABS078	328	Iran	wound	clinical	2013	SAMN12726219	GCA_009497535.1	Contig	4.18394	38.8		WIOD01	97	97	127074	abaF=COMPLETE,amvA=COMPLETE,ant(2'')-Ia=COMPLETE,ant(3'')-IIa=COMPLETE,aph(3')-VIa=COMPLETE,blaADC=COMPLETE,blaOXA-23=COMPLETE,blaOXA-69=COMPLETE,blaOXA-72=COMPLETE,sul2=COMPLETE
ABS103	328	Iran	wound	clinical	2013	SAMN12726220	GCA_009497515.1	Contig	4.24477	38.8		WIOC01	107	107	150145	abaF=COMPLETE,amvA=COMPLETE,ant(2'')-Ia=COMPLETE,ant(3'')-IIa=COMPLETE,aph(3')-VIa=COMPLETE,blaADC=COMPLETE,blaOXA-23=COMPLETE,blaOXA-69=COMPLETE,blaOXA-72=COMPLETE,sul2=COMPLETE
ABS104	328	Iran	blood	clinical	2013	SAMN12726221	GCA_009497505.1	Contig	4.13683	38.8		WIOB01	102	102	150144	abaF=COMPLETE,amvA=COMPLETE,ant(2'')-Ia=COMPLETE,ant(3'')-IIa=COMPLETE,aph(3')-VIa=COMPLETE,blaADC=COMPLETE,blaOXA-23=COMPLETE,blaOXA-69=COMPLETE,blaOXA-72=COMPLETE,sul2=COMPLETE
ABS122	328	Iran	wound	clinical	2013	SAMN12726222	GCA_009497435.1	Contig	4.10255	38.9		WIOA01	108	108	102004	abaF=COMPLETE,amvA=COMPLETE,ant(3'')-IIa=COMPLETE,aph(3')-VIa=COMPLETE,blaADC=COMPLETE,blaOXA-23=COMPLETE,blaOXA-69=COMPLETE,blaOXA-72=COMPLETE,sul2=COMPLETE,tet(39)=COMPLETE
AC19	1	Malaysia	ETT secretion	clinical	2011	SAMN12810223	GCA_008806495.1	Contig	4.01606	39.1		VZZN01	55	55	226206	aac(3)-Ia=COMPLETE,aadA1=COMPLETE,abaF=COMPLETE,adeC=COMPLETE,amvA=COMPLETE,ant(3'')-IIa=COMPLETE,aph(3')-Ia=COMPLETE,blaADC=PARTIAL_END_OF_CONTIG,blaOXA-23=COMPLETE,blaOXA-69=COMPLETE,blaTEM=PARTIAL,catA1=COMPLETE,mph(E)=COMPLETE,msr(E)=COMPLETE,sul1=COMPLETE,tet(A)=COMPLETE
FK_2016_46	1	Greece	urine	clinical	2016	SAMN13066399	GCA_009760475.1	Scaffold	3.94036	38.9		WIHP01	55	58	137638	aac(3)-Ia=COMPLETE,aadA1=PARTIAL,abaF=COMPLETE,adeC=COMPLETE,amvA=COMPLETE,ant(3'')-IIa=COMPLETE,aph(3')-VIa=COMPLETE,blaADC-175=COMPLETE,blaOXA-23=COMPLETE,blaOXA-69=COMPLETE,sul1=COMPLETE
FK_2016_47	1	Greece	sputum	clinical	2016	SAMN13066400	GCA_009760485.1	Scaffold	3.97252	38.9		WIHO01	52	53	145751	aac(3)-Ia=COMPLETE,aadA1=PARTIAL,abaF=COMPLETE,adeC=COMPLETE,amvA=COMPLETE,ant(3'')-IIa=COMPLETE,aph(3')-VIa=COMPLETE,blaADC-175=COMPLETE,blaOXA-23=COMPLETE,blaOXA-69=COMPLETE,sul1=COMPLETE
SI_2017_65	1	Greece	blood	clinical	2017	SAMN13066415	GCA_009759915.1	Scaffold	3.97329	38.9		WIGZ01	61	65	142589	aac(3)-Ia=COMPLETE,aadA1=PARTIAL,abaF=COMPLETE,adeC=COMPLETE,amvA=COMPLETE,ant(3'')-IIa=COMPLETE,aph(3')-VIa=COMPLETE,blaADC-175=COMPLETE,blaOXA-23=COMPLETE,blaOXA-69=COMPLETE,sul1=COMPLETE
ACI40	1	Brazil: Belo Horizonte	tracheal aspirate	clinical	8/22/16	SAMN13157208	GCA_013138475.1	Scaffold	3.9464	39		WJWR01	81	84	96643	aac(6')-Ib=COMPLETE,abaF=COMPLETE,adeC=COMPLETE,amvA=COMPLETE,ant(3'')-IIa=COMPLETE,aph(3'')-Ib=COMPLETE,aph(3')-IIa=COMPLETE,aph(6)-Id=COMPLETE,blaADC-181=COMPLETE,blaOXA-23=COMPLETE,blaOXA-69=COMPLETE,blaTEM=COMPLETE,floR=COMPLETE,sul2=COMPLETE
ACI42	1	Brazil: Belo Horizonte	tracheal aspirate	clinical	8/30/16	SAMN13160111	GCA_013138415.1	Scaffold	3.9452	39.1		WJWS01	185	199	50175	aac(6')-Ib=COMPLETE,abaF=COMPLETE,adeC=COMPLETE,amvA=COMPLETE,ant(3'')-IIa=COMPLETE,aph(3'')-Ib=COMPLETE,aph(3')-IIa=COMPLETE,aph(6)-Id=COMPLETE,blaADC-181=COMPLETE,blaOXA-23=COMPLETE,blaOXA-69=COMPLETE,blaTEM-116=COMPLETE,floR=COMPLETE,sul2=COMPLETE
ACI53	1	Brazil: Belo Horizonte	urine	clinical	8/26/16	SAMN13160208	GCA_013138435.1	Scaffold	3.94724	38.9		WJWT01	98	100	73907	aac(6')-Ib=COMPLETE,abaF=COMPLETE,adeC=COMPLETE,amvA=COMPLETE,ant(3'')-IIa=COMPLETE,aph(3'')-Ib=COMPLETE,aph(3')-IIa=COMPLETE,aph(6)-Id=COMPLETE,blaADC-181=COMPLETE,blaOXA-23=COMPLETE,blaOXA-69=COMPLETE,blaTEM-116=COMPLETE,floR=COMPLETE,mph(E)=COMPLETE,msr(E)=COMPLETE,sul2=COMPLETE,tet(39)=COMPLETE
A-71.193	1	Brazil	blood	clinical	2016	SAMN13622373	GCA_009829545.1	Contig	3.80335	38.9		WTZL01	53	53	136455	aac(6')-Ib=COMPLETE,adeC=COMPLETE,amvA=COMPLETE,ant(3'')-IIa=COMPLETE,aph(3')-VIa=COMPLETE,blaADC-235=COMPLETE,blaOXA-23=COMPLETE,blaOXA-69=COMPLETE,blaOXA-72=COMPLETE,mph(E)=COMPLETE,msr(E)=COMPLETE
VNMU81	19	Ukraine	wound	clinical	3/23/15	SAMN13701828	GCA_009901675.1	Contig	4.00335	38.9		WYAS01	100	100	110265	abaF=COMPLETE,adeC=COMPLETE,amvA=COMPLETE,ant(2'')-Ia=COMPLETE,ant(3'')-IIa=COMPLETE,aph(3')-VIa=COMPLETE,blaADC=COMPLETE,blaOXA-69=COMPLETE,catA1=COMPLETE,sul2=COMPLETE,tet(B)=COMPLETE
VNMU85	19	Ukraine	wound	clinical	3/23/15	SAMN13701829	GCA_009901705.1	Contig	3.81907	38.9		WYAR01	83	83	110265	adeC=COMPLETE,amvA=COMPLETE,ant(2'')-Ia=COMPLETE,ant(3'')-IIa=COMPLETE,blaADC=COMPLETE,blaOXA-69=COMPLETE,sul2=COMPLETE
VNMU133	1	Ukraine	wound	clinical	10/16/16	SAMN13701832	GCA_009887905.1	Contig	4.06062	38.9		WYAO01	61	61	216107	aac(3)-Ia=COMPLETE,aadA1=COMPLETE,aadA2=COMPLETE,abaF=COMPLETE,adeC=COMPLETE,amvA=COMPLETE,ant(3'')-IIa=COMPLETE,aph(3')-VIa=COMPLETE,armA=COMPLETE,blaADC=COMPLETE,blaOXA-23=COMPLETE,blaOXA-69=COMPLETE,blaTEM-1=COMPLETE,dfrA12=COMPLETE,mph(E)=MISTRANSLATION,msr(E)=COMPLETE,sul1=COMPLETE
VNMU134	19	Ukraine	wound	clinical	11/11/16	SAMN13701833	GCA_009887785.1	Contig	3.99666	38.9		WYAN01	107	107	89679	abaF=COMPLETE,adeC=COMPLETE,amvA=COMPLETE,ant(2'')-Ia=PARTIAL_END_OF_CONTIG,ant(3'')-IIa=COMPLETE,aph(3')-Ia=PARTIAL_END_OF_CONTIG,blaADC=COMPLETE,blaOXA-69=COMPLETE,catA1=COMPLETE,sul2=COMPLETE
SP2486	1	India: Vellore	Endotracheal Aspirate	clinical	2019	SAMN14001769	GCA_010500435.1	Contig	4.0462	38.8		JAAGTH01	169	169	74360	abaF=COMPLETE,adeC=COMPLETE,amvA=COMPLETE,ant(3'')-IIa=COMPLETE,aph(3'')-Ib=COMPLETE,aph(3')-VIa=COMPLETE,aph(6)-Id=COMPLETE,blaADC=COMPLETE,blaNDM-1=COMPLETE,blaOXA-23=COMPLETE,blaOXA-371=COMPLETE,ble=COMPLETE,mph(E)=COMPLETE,msr(E)=COMPLETE,sul2=COMPLETE
SP3561	1	India: Vellore	Endotracheal Aspirate	clinical	2019	SAMN14299634	GCA_011603925.1	Contig	3.96429	39.1		JAAOSU01	74	74	134026	aac(3)-Ia=COMPLETE,aadA1=PARTIAL_END_OF_CONTIG,abaF=COMPLETE,adeC=COMPLETE,amvA=COMPLETE,ant(3'')-IIa=COMPLETE,aph(3'')-Ib=COMPLETE,aph(3')-Ia=COMPLETE,aph(3')-VIa=COMPLETE,aph(6)-Id=PARTIAL_END_OF_CONTIG,blaADC-191=COMPLETE,blaNDM=COMPLETE,blaOXA-23=COMPLETE,blaOXA-69=COMPLETE,ble=COMPLETE,dfrA1=COMPLETE
SP4673	1	India: Vellore	Endotracheal Aspirate	clinical	2019	SAMN14299653	GCA_011603485.1	Contig	3.96445	39.1		JAAOSB01	70	70	148061	aac(3)-Ia=COMPLETE,aadA1=PARTIAL_END_OF_CONTIG,abaF=COMPLETE,adeC=COMPLETE,amvA=COMPLETE,ant(3'')-IIa=COMPLETE,aph(3'')-Ib=COMPLETE,aph(3')-Ia=COMPLETE,aph(3')-VIa=COMPLETE,aph(6)-Id=COMPLETE,blaADC-191=COMPLETE,blaNDM=COMPLETE,blaOXA-23=COMPLETE,blaOXA-69=COMPLETE,ble=COMPLETE,dfrA1=COMPLETE,sat2=COMPLET
VB2486	1	India	sputum	clinical	2019	SAMN14414761	GCA_012935005.1	Complete	4.27787	39.172	chromosome:NZ_CP05040	7		7	4137731	abaF=COMPLETE,adeC=COMPLETE,amvA=COMPLETE,ant(3'')-IIa=COMPLETE,aph(3'')-Ib=COMPLETE,aph(3')-VIa=COMPLETE,aph(6)-Id=COMPLETE,blaADC=COMPLETE,blaNDM-1=COMPLETE,blaOXA-23=COMPLETE,blaOXA-371=COMPLETE,ble=COMPLETE,mph(E)=COMPLETE,msr(E)=COMPLETE,sul2=COMPLETE
Aba 18S	1	Romania:Children Hospita	tracheal secretion	clinical	9/17/17	SAMN14525746	GCA_013185135.1	Scaffold	3.97857	39.1		JAAWWU01	173	177	81560	aac(3)-Ia=COMPLETE,aac(6')-Ib3=COMPLETE,aadA1=COMPLETE,abaF=COMPLETE,adeC=COMPLETE,amvA=COMPLETE,ant(3'')-IIa=COMPLETE,aph(3')-VIa=COMPLETE,blaADC-81=COMPLETE,blaOXA-72=COMPLETE,blaOXA-92=COMPLETE,blaTEM-1=COMPLETE,catA1=COMPLETE,sul1=PARTIAL_END_OF_CONTIG,tet(A)=COMPLETE
AB49	1	Brazil:Roraima	catheter tip	clinical	1/3/17	SAMN14549413	GCA_012276735.1	Contig	3.94084	39.1		JAAXKW01	77	77	210324	aac(3)-Ia=COMPLETE,aac(6')-Ib=COMPLETE,abaF=COMPLETE,adeC=COMPLETE,amvA=COMPLETE,ant(3'')-IIa=COMPLETE,aph(3')-Ia=COMPLETE,blaADC-182=COMPLETE,blaOXA-23=COMPLETE,blaOXA-69=COMPLETE,sul1=COMPLETE
A7-T	1	Egypt: Alexandria		clinical	2015	SAMN15915097	GCA_014531735.1	Contig	4.12626	39		JACSVP01	93	93	186853	aac(3)-Ia=COMPLETE,aac(6')-Ib3=COMPLETE,aadA1=COMPLETE,aadA2=COMPLETE,abaF=COMPLETE,adeC=COMPLETE,amvA=COMPLETE,ant(2'')-Ia=COMPLETE,ant(3'')-IIa=COMPLETE,aph(3'')-Ib=COMPLETE,aph(3')-Ia=COMPLETE,aph(3')-VIa=COMPLETE,aph(6)-Id=COMPLETE,blaADC-11=COMPLETE,blaGES-11=COMPLETE,blaOXA-23=COMPLETE,blaOXA-69=COMPLETE,
A8-T	1	Egypt: Alexandria		clinical	2015	SAMN15915098	GCA_014531705.1	Contig	4.1241	39.1		JACSVO01	99	99	285515	aac(3)-Ia=COMPLETE,aac(6')-Ib3=COMPLETE,aadA1=COMPLETE,aadA2=COMPLETE,abaF=COMPLETE,adeC=COMPLETE,amvA=COMPLETE,ant(2'')-Ia=COMPLETE,ant(3'')-IIa=COMPLETE,aph(3'')-Ib=COMPLETE,aph(3')-Ia=COMPLETE,aph(3')-VIa=COMPLETE,aph(6)-Id=COMPLETE,blaADC-11=COMPLETE,blaGES-11=COMPLETE,blaOXA-23=COMPLETE,blaOXA-69=COMPLETE,
A18	19	Egypt: Alexandria		clinical	2015	SAMN15915108	GCA_014531335.1	Contig	4.02949	39		JACSTP01	243	243	48652	aac(6')-Ib3=COMPLETE,adeC=COMPLETE,amvA=COMPLETE,ant(2'')-Ia=COMPLETE,ant(3'')-IIa=COMPLETE,aph(3')-Ia=COMPLETE,aph(3')-VIa=COMPLETE,blaADC-79=COMPLETE,blaGES-12=COMPLETE,blaOXA-69=COMPLETE,blaOXA=PARTIAL_END_OF_CONTIG,catA1=COMPLETE,dfrA7=COMPLETE,sul1=PARTIAL_END_OF_CONTIG,sul2=COMPLETE
A35	1	Egypt: Alexandria		clinical	2010	SAMN15915115	GCA_014531135.1	Contig	3.98369	38.9		JACSTJ01	239	239	38052	aac(6')-Ib3=COMPLETE,abaF=COMPLETE,adeC=COMPLETE,amvA=COMPLETE,ant(3'')-IIa=COMPLETE,blaADC-11=COMPLETE,blaGES-11=COMPLETE,blaOXA-23=COMPLETE,blaOXA-69=COMPLETE,dfrA7=COMPLETE
A87	1	Egypt: Alexandria		clinical	2010	SAMN15915140	GCA_014530695.1	Contig	4.11379	39		JACSSN01	194	194	114219	aac(3)-Ia=COMPLETE,aac(6')-Ib3=COMPLETE,aadA1=COMPLETE,abaF=COMPLETE,adeC=COMPLETE,amvA=COMPLETE,ant(3'')-IIa=COMPLETE,aph(3')-Ia=COMPLETE,aph(3')-VIa=COMPLETE,blaADC-11=COMPLETE,blaGES=PARTIAL_END_OF_CONTIG,blaOXA-23=COMPLETE,blaOXA-69=COMPLETE,blaTEM-1=COMPLETE,catA1=COMPLETE,dfrA7=COMPLETE,sul1=COMPLET
Ax270	1	Lebanon	cat rectal swab	environmental/other	2015	SAMN14149266		complete			chromosom	CP049240				
Ex003	1	Lebanon	water; artesian well	environmental/other	2012	SAMN14149265		complete			chromosom	CP049314				

Table S3

Assembly	Strain	K locus	OC locus
SAMEA104411068	ACB5	KL17	OCL1
SAMEA1317924	D2	KL1b	OCL1
SAMEA1317930	D3208	KL1	OCL1
SAMEA1317939	WM98	KL1a	OCL1
SAMEA1466011	A92	KL1	OCL1
SAMEA1466028	G7	KL17	OCL1
SAMEA1466056	D30	KL1	OCL1
SAMEA1466082	A83	KL1	OCL1
SAMEA1709744	J1	KL1	OCL1
SAMEA1709749	A297	KL1	OCL1
SAMEA1709843	J10	KL1a	OCL1
SAMEA1709848	J7	KL1a	OCL1
SAMEA1709886	J5	KL1	OCL1
SAMEA1876472	Ab118	KL1b	OCL1
SAMEA3138279	AYE	KL1	OCL1
SAMEA3296319	CHI-45-1	KL4	OCL1
SAMEA3298507	CHI-34	KL4	OCL1
SAMEA5603549	aba_5m	KL17	OCL1
SAMEA5987517	Aci00879	KL17	OCL1
SAMEA5987518	Aci00880	KL17	OCL1
SAMEA5987519	Aci00881	KL17	OCL1
SAMEA5987520	Aci00882	KL17	OCL1
SAMN00761238	Naval-83	KL1c	OCL1
SAMN00761242	Naval-21	KL15	OCL1
SAMN01828143	NIPH 290	KL1	OCL1
SAMN01828150	NIPH 527	KL1	OCL1
SAMN02437318	MRSN 57	KL1	OCL1
SAMN02437332	MRSN 58	KL1	OCL1
SAMN02470682	ABNIH11	KL1*	OCL1
SAMN02470684	ABNIH19	KL1*	OCL1
SAMN02470695	ABNIH6	KL1*	OCL1
SAMN02906928	MRSN 58	KL1	OCL1
SAMN03078661	AB3340	KL1c	OCL1
SAMN03078682	AB5197	KL1	OCL1
SAMN03248539	A1	KL1	OCL1
SAMN03276491	A155	KL1	OCL1
SAMN04014924	AR_0083	KL17	OCL1
SAMN04272864	AC001	KL1c	OCL1
SAMN04272867	AC001-8-R1	KL1c	OCL1
SAMN04272868	AC001-8-R1-	KL1c	OCL1
SAMN04407353	MEX11594	KL1	OCL1
SAMN05229953	CRO1	KL42a	OCL1
SAMN05229955	CRO3	KL17a	OCL1
SAMN05590239	71	KL4	OCL1
SAMN05590405	440	KL4	OCL1
SAMN07257378	13	KL1*	OCL1
SAMN07286190	15	KL18*	OCL1
SAMN07303764	IHSS3526	KL146	OCL1
SAMN07514514	Abau446	KL17*	OCL1
SAMN07961492	AB307-0294	KL1	OCL1
SAMN08093364	ZQ5	KL17	OCL1
SAMN08364584	WCHAB0050	KL1	OCL1
SAMN08383932	A1-360	KL1	OCL1
SAMN08383996	E1-143	KL1	OCL1
SAMN10797507	521_17	KL18a	OCL1
SAMN12015779	11W359501	KL17	OCL1
SAMN12087645	MRSN14193	KL146	OCL1
SAMN12087664	MRSN31196	KL1*	OCL1
SAMN12087696	MRSN960	KL1*	OCL1
SAMN12087708	MRSN6541	KL1*	OCL1
SAMN12087714	MRSN7213	KL1	OCL1
SAMN13160208	ACI53	KL18a	OCL1
SAMN13622373	A-71.193	KL18*	OCL1

SAMN13701832	VNMU133	KL1	OCL1
SAMN14299634	SP3561	KL17	OCL1
SAMN14299653	SP4673	KL17	OCL1
SAMN14549413	AB49	KL17	OCL1
Ax270	outgroup	KL1	OCL1
Ex003		KL1	OCL1
SAMN08093365	ZQ6	KL17	OCL1::TnAphA6
SAMEA1317920	D81	KL4	OCL1b
SAMEA1317937	D78	KL4	OCL1b
SAMN13157208	ACI40	KL18a*	OCL1r
SAMN13160111	ACI42	KL18a	OCL1r
SAMEA3724634	B	KL40*	OCL2
SAMEA3724638	F	KL40	OCL2
SAMEA3724639	G	KL40	OCL2
SAMEA3724640	I	KL40	OCL2
SAMEA3724641	J	KL40	OCL2
SAMEA3724642	K	KL40	OCL2
SAMN00189090	6013113	KL12*	OCL2*
SAMN00189091	6013150	KL12*	OCL2
SAMN00761215	IS-58	KL25	OCL2
SAMN00761221	IS-235	KL25*	OCL2
SAMN00761222	IS-251	KL25	OCL2
SAMN01828141	ANC 4097	KL40	OCL2
SAMN02628532	R1B	KL4	OCL2
SAMN02713669	AB5075	KL25	OCL2
SAMN02894434	AB5075-UW	KL25	OCL2
SAMN02906923	MRSN 3527	KL12	OCL2
SAMN03078672	AB4448	KL25*	OCL2
SAMN03078674	AB4490	KL25*	OCL2
SAMN03078683	AB5674	KL25	OCL2
SAMN04029125	D36	KL12	OCL2
SAMN06837753	PR332	KL12	OCL2
SAMN09460321	DA33382	KL40	OCL2
SAMN11626014	ABS201	KL18	OCL2
SAMN12087641	MRSN15049	KL12	OCL2*
SAMN12087695	MRSN959	KL25	OCL2
SAMN12087709	MRSN7067	KL12	OCL2
SAMN12726215	ABS029	KL18	OCL2
SAMN12726217	ABS062	KL18	OCL2
SAMN12726218	ABS063	KL18	OCL2
SAMN12726222	ABS122	KL18	OCL2
SAMN13066399	FK_2016_46	KL40	OCL2
SAMN13066400	FK_2016_47	KL40	OCL2
SAMN13066415	SI_2017_65	KL40	OCL2
SAMN15915097	A7-T	KL1d	OCL2
SAMN15915098	A8-T	KL1e	OCL2
SAMN15915140	A87	KL17*	OCL2
SAMN12726216	ABS042	KL13	OCL2a
SAMN12726219	ABS078	KL13	OCL2a
SAMN12726220	ABS103	KL13	OCL2a
SAMN12726221	ABS104	KL13	OCL2a
SAMN15915115	A35	KL40a	OCL2b
SAMEA3724644	M	KL40*	OCL2c
SAMEA1317927	RBH3	KL15	OCL3
SAMEA1465988	6772166	KL15	OCL3
SAMEA1466052	A85	KL15	OCL3
SAMEA3146530	NCTC13421	KL4	OCL3
SAMEA5396111	Aci00872	KL18*	OCL3
SAMN00761234	Canada BC1	KL15	OCL3
SAMN02055511	TG20277	KL15	OCL3
SAMN02055516	TG22112	KL4	OCL3
SAMN02055519	TG22148	KL4	OCL3
SAMN02055521	TG22190	KL4	OCL3
SAMN02055523	TG22194	KL4	OCL3
SAMN02055524	TG22196	KL4	OCL3
SAMN02055528	TG22214	KL4	OCL3

SAMN02436468	Canada BC-5	KL15	OCL3
SAMN02436567	1605	KL15	OCL3
SAMN02471253	AB_908-13	KL4	OCL3
SAMN02471282	AB_909-02-7	KL4	OCL3
SAMN02603051	AB0057	KL4	OCL3
SAMN02906929	MRSN 7339	KL15	OCL3
SAMN03078666	AB3927	KL18	OCL3
SAMN03078680	AB4991	KL4	OCL3
SAMN03105183	6870155	KL15	OCL3
SAMN03418031	S36	KL107	OCL3
SAMN04014886	AR_0045	KL4	OCL3
SAMN06892213	ARLG1314	KL4	OCL3
SAMN07125723	A85	KL15	OCL3
SAMN08637734	SGH0410	KL4	OCL3
SAMN08637735	SGH0411	KL4	OCL3
SAMN08637736	SGH0606	KL107	OCL3
SAMN08637738	SGH0807	KL70	OCL3
SAMN08637739	SGH0915	KL17	OCL3
SAMN10249015	AB_235	KL12	OCL3
SAMN10261541	TG22592	KL4	OCL3
SAMN10261559	TG29424	KL4	OCL3
SAMN10261577	TG22636	KL4	OCL3
SAMN10261598	TG15482	KL4	OCL3
SAMN12087674	MRSN32108	KL18	OCL3
SAMN12087702	MRSN1311	KL4	OCL3
SAMN12810223	AC19	KL4	OCL3
SAMN06650240	USA15	KL40	OCL3b
SAMEA1466008	A388	KL20	OCL4
SAMN07736509	A388	KL20	OCL4
SAMN14525746	Aba 18S	KL1	OCL4
SAMEA1317922	D13	KL1	OCL5
SAMEA3139099	Pulsotype 27	KL15	OCL5*
SAMEA4646215	KCRI-363	KL18	OCL5
SAMEA4646216	KCRI-423	KL18	OCL5
SAMEA4646220	KCRI-518B	KL18	OCL5
SAMEA4646221	KCRI-558	KL18	OCL5
SAMEA5396089	Aci00834	KL91*	OCL5*
SAMN00761203	OIFC074	KL1	OCL5
SAMN08093362	ZQ3	KL12	OCL5
SAMN08637740	SGH1101	KL4	OCL5
SAMN09244198	5457	KL91	OCL5
SAMN10170275	VB29123	KL17	OCL5
SAMN10338982	VB24319	KL17	OCL5
SAMN12087678	MRSN32865	KL125	OCL5
SAMN12509150	2846	KL91	OCL5
SAMN13701828	VNMU81	KL91	OCL5
SAMN13701833	VNMU134	KL91*	OCL5
SAMN14001769	SP2486	KL17*	OCL5
SAMN14414761	VB2486	KL17b	OCL5
SAMN15915108	A18	KL91	OCL5*
SAMEA6436546	AC3-M	KL91	OCL5*
SAMN04254727	MRSN4119	KL15	OCL5*
SAMN02836944	MRSN 3405	KL15	OCL5e*
SAMN02906922	MRSN 3405	KL15	OCL5e
SAMN02906926	MRSN 3942	KL15	OCL5e
SAMN04254728	MRSN3941	KL15	OCL5e
SAMN04254729	MRSN5540	KL15	OCL5e
SAMN04254730	MRSN6269	KL15	OCL5e
SAMN04254731	MRSN6273	KL15*	OCL5e
SAMN02906927	MRSN 4106	KL15	OCL5f*
SAMN12087687	MRSN48967:	KL147	OCL5g
SAMN13701829	VNMU85	KL147	OCL5g
SAMN07520232	9102	KL1	OCL7

* = Gene cluster found broken across 2 or more contigs
